# Bromo‐Heptahelicene‐Bis‐Thiadiazole: Photophysics, Chiroptics, and Excited‐State Dynamics

**DOI:** 10.1002/cphc.202500176

**Published:** 2025-05-12

**Authors:** Debsouri Kundu, Rituparno Chowdhury, Natalia Del Rio, Marie Cordier, Nicolas Vanthuyne, Richard H. Friend, Monika Srebro‐Hooper, Jeanne Crassous

**Affiliations:** ^1^ Institut des Sciences Chimiques de Rennes Univ Rennes UMR CNRS 6226 Campus de Beaulieu 35042 Rennes Cedex France; ^2^ The Cavendish Laboratory University of Cambridge J. J. Thomson Avenue Cambridge CB3 0HE UK; ^3^ Aix Marseille Univ CNRS Centrale Med FSCM 13397 Marseille France; ^4^ Faculty of Chemistry Jagiellonian University Gronostajowa 2 30‐387 Krakow Poland

**Keywords:** chiroptics, heavy‐atom effects, heptahelicenes, photophysics, time‐dependent density functional theory calculations

## Abstract

The synthesis of a [7]helicene bromide derivative with two fused 2,1,3‐thiadiazole heterocycles (**TD[7]Br**) and a comprehensive study of its photophysical and chiroptical characteristics are presented along with a comparison with 9‐bromo‐carbo[7]helicene (**[7]Br**) and 2,15‐dibromo‐carbo[6]helicene (**[6]Br**). The integration of a bromine heavy atom onto the helicene backbone facilitates efficient singlet‐to‐triplet conversion allowing to investigate the resulting fluorescence and phosphorescence properties. The steady‐state chiroptical features of the systems are demonstrated through electronic circular dichroism and circularly polarized luminescence. Interestingly, a fluorescence quantum yield of 14% is obtained, a 17‐fold increase compared to the corresponding bromo‐heptacarbohelicene, and phosphorescence dissymmetry factors reach ±1.2 × 10^−2^ at 580 nm at low temperature. Finally, the exploration of various excited states generated during the excitation process and their dynamics is delved into by employing nonpolarized transient absorption and emission spectroscopies, thus, highlighting the fruitful combination of heavy‐atom effect and charge transfer. The experimental results are understood through time‐dependent density functional theory computations.

## Graphical abstract

The substitution of a heptahelicene‐bis‐thiadiazole molecule with a bromine heavy atom leads to an increased luminescence quantum yield at room temperature and enhanced circularly polarized luminescence activity at low temperature. Time‐dependent photophysical analyses and theoretical calculations enable to highlight contributions from direct and reverse singlet–triplet intersystem crossing processes.

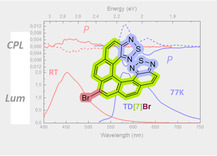

## 1 Introduction

**Figure 1:**
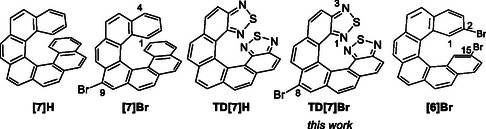
Heptahelicenic architectures bearing a bromine atom (**[7]Br**) or not (**[7]H**) at 9 position along with their corresponding bis‐thiadiazole derivatives **TD[7]H** and **TD[7]Br** (described in this work). On the right, the chemical structure of 2,15‐bis‐bromo[6]helicene (**[6]Br**) is presented.

Helicenes, formed of *ortho*‐fused aromatic rings, have become a well‐known class of helical polycyclic aromatic hydrocarbons, thanks to their unique topology and extended *π*‐conjugation, resulting in appealing photophysical and chiroptical properties.^[^ ^1^ ^]^ They have found potential applications in diverse areas, such as asymmetric catalysis, molecular recognition, chiral bioimaging, spin filtering, or as chiral emissive dopants in circularly polarized (CP) organic light‐emissive diodes.^[^ ^2^ ^]^ In order to improve the efficiencies of these devices and fine tune the desired properties, understanding the photophysical properties of helicenes upon varying the molecular structure is of utmost importance. Parent carbo[7]helicene (**[7]H**, **Figure** **1**) demonstrates a low fluorescence quantum yield (*Φ*_f_) of 2% and a modest CP luminescence (CPL) activity with a dissymmetry factor (*g*_lum_) of +6× 10^−3^ in chloroform for the (*P*)‐isomer.^[^ ^3^ ^]^ Heteroatoms substitution into the helical core or their introduction as external substituents has been shown to significantly impact the optical properties of heptahelicenes.^[^ ^4^ ^]^ For example, (benzo)thiadiazole heterocycles, which have been widely used in the development of conjugated materials for light‐emitting devices, organic field‐effect transistors, and photovoltaic devices, have been successfully incorporated onto helicene backbone,^[^ ^5^ ^]^ leading to appealing applications, including emission properties^[^ ^6^ ^]^ and oxygen evolution reaction mediated by the chiral‐induced spin selectivity effect.^[^ ^7^ ^]^ Recently, [7]helicene fused with two terminal 2,1,3‐thiadiazole rings (**TD[7]H**) has been synthesized by Hirose and co‐workers, showing a high *g*_lum_ value of 1 × 10^−2^ but a *Φ*_f_ value of only 0.7%.^[^ ^8^ ^]^ In the present work, we demonstrated that introducing a bromine heavy atom at position 8 of **TD[7]H** (leading to **TD[7]Br**) significantly improves the radiative processes including fluorescence quantum yield while maintaining its CPL activity. To understand the potential structure–property relationships in these systems, we synthesized analogous brominated carbohelicene molecules, 9‐bromo‐carbo[7]helicene (**[7]Br**) and bis‐brominated 2,15‐dibromo‐carbo[6]helicene (**[6]Br**). The obtained results were analyzed with the help of transient spectroscopic studies and detailed theoretical calculations. The work highlights the benefit of combining heavy‐atom effects with molecular donor–acceptor characteristics.

## 2 Results and Discussion

### 2.1 Synthesis

**Scheme 1:**
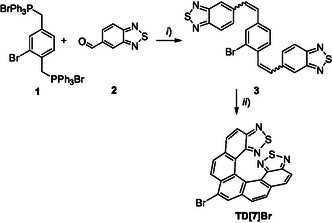
Synthesis of **TD[7]Br**: *i*) *n*‐BuLi, THF, rt, Ar, 3 h, 40%; *ii*) toluene, hν, I_2_ (2 eq.), propylene oxide (20 eq.), 18 h, 90%.

Inspired by documented methods from Avarvari and co‐workers,^[^ ^5^, ^6^, ^7^ ^]^ we synthesized bromo‐[7]helicene‐bis‐thiadiazole, denoted as **TD[7]Br**, following a protocol depicted in **Scheme** **1**.

The synthetic scheme started with bis‐phosphonium salt **1**, which was prepared as previously reported by Katz.^[^ ^9^ ^]^ Then, a Wittig reaction between **1** and 5‐formyl‐2,1,3‐benzothiadiazole (**2**) produced the corresponding bis‐alkene **3**, as a mixture of *cis*/*cis*‐, *cis*/*trans*‐, and *trans*/*trans*‐isomers in 40% yield. The Mallory photocyclization reaction^[^ ^10^ ^]^ of this isomeric mixture of bis‐olefins was then performed under classical conditions (see the Supporting Information), leading to **TD[7]Br** with a very efficient yield of 90%. Detailed spectroscopic and structural characterizations of **TD[7]Br** are reported in the Supporting Information. It is crucial to highlight the influential role of the bromine atom in directing the photocyclization process. Indeed, when subjected to analogous photocyclization reactions without bromine substituent, **TD[7]H** was obtained with yields of around 40–45% with an equimolar formation of an S‐shaped regioisomer.^[^ ^6^, ^11^ ^]^ Thus, the presence of bromine imposes the cyclization toward the *ortho*‐fused heptahelicenic system. Using a similar methodology, the Wittig reaction of 2‐naphthaldehyde with bis‐phosphonium salt **1**, followed by the Mallory photocyclization reaction, resulted in the formation of [7]helicene denoted as **[7]Br**.^[^ ^11^ ^]^ Enantiomeric resolutions of racemic compounds **TD[7]Br** and **[7]Br** were conducted by chiral high‐performance liquid chromatography (HPLC) separation techniques, yielding pure enantiomers (enantiomeric excesses *ee*'s, higher than 99.5%, see the Supporting Information), while the enantiopure dibromohelicene samples, that is, (*P*)‐ and (*M*)‐**[6]Br**, were prepared as already reported.^[^ ^12^ ^]^

### 2.2 Steady‐State Photophysical Properties

**Figure 2:**
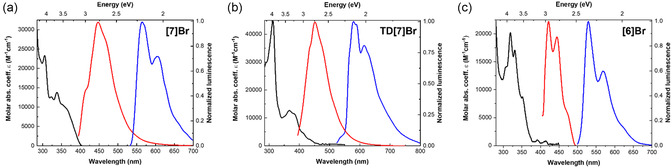
UV–vis absorption (black line) along with normalized emission in toluene at 298 K (red line) and in 2‐MeTHF at 77 K (blue line) spectra for a) **[7]Br** (*C* 1 × 10^−5^ m, *λ*_exc_ 365 nm), b) **TD[7]Br** (*C* 5 × 10^−6^ m, *λ*_exc_ 385 nm), and c) **[6]Br** (*C* 1 × 10^−5^ m, *λ*_exc_ 365 nm).

The heavy‐atom effect is known to facilitate singlet–triplet conversion and to enhance the resulting phosphorescence properties of a molecule. This phenomenon arises from the spin–orbit coupling (SOC) between the excited‐state electrons and the large nucleus of the heavy atom.^[^ ^13^ ^]^ It is thus of interest to study the influence of bromine on the photophysical properties of helicene‐based molecules. The absorption and emission spectra of **TD[7]Br**, **[7]Br**, and **[6]Br** were measured in toluene at room temperature (298 K) and in 2‐methyltetrahydrofuran (2‐MeTHF) at cryogenic temperature (77 K), as shown in **Figure** **2** and listed in **Table** **1**. As shown in Figure 2a, **[7]Br** exhibits a significant band at 305 nm with a molar extinction coefficient (*ε*) of 23,500 m ^−1^ cm^−1^ in the higher‐energy region and a moderate absorption band in the range of 315–350 nm (*ε* ≈ 12,800 m ^−1^ cm^−1^ at 322 nm and *ε* ≈ 13,900 m ^−1^ cm^−1^ at 337 nm) with a shoulder at 365 nm (*ε* ≈ 9400 m ^−1^ cm^−1^). In comparison, pristine **[7]H** was reported^[^ ^3^ ^]^ to exhibit a very weak absorption band in chloroform at 423 nm (*ε* = 200 m ^−1^ cm^−1^) but a very strong band between 250 and 300 nm of *ε* > 80,000 m ^−1^ cm^−1^.

**Table 1:** Photophysical properties of **[7]Br**, **TD[7]Br**, and **[6]Br** helicenes under the conditions indicated. Lifetimes are derived using a multi‐exponential fitting model, percentages of each component are denoted in parentheses. Radiative and nonradiative rate constants are calculated based on the main lifetime component (>90% contribution).

Compound	Absorption in toluene at 298 K *λ*_max_ [nm] (*ε* [10^3^ M^−1^ cm^−1^])	Emission in toluene at 298 K					Emission in 2‐MeTHF at 77 K *λ*_max_ [nm]
		*λ*_max_ [nm]	*Φ* _lum_ [%]	*τ* [ns]a)	*k*_r_ [s^−1^]	*k*_nr_ [s^−1^]	
**[7]Br**	305 (23.5), 322 (12.8), 337 (13.9), 365 (9.4)	447, 411	0.8	0.3 (97.9%), 1.6 (1.8%), 16 (0.3%)	2.7 × 10^7^	3.3 × 10^9^	564, 605
**TD[7]Br**	313 (44.8), 366 (12.8), 385 (11.6)	451	14	0.4 (90%), 1.9 (10%)	3.5 × 10^8^	2.15 × 10^9^	580, 614
**[6]Br**	319 (20.4), 391 (1.04), 415 (0.98)	421, 444, 473	0.6	0.2 (98.9%), 2.9 (1.1%)	3.0 × 10^7^	5.0 × 10^9^	528, 574, 621

a) The decays do not fit to a single‐exponential decay function; the quoted values are amplitude‐weighted average lifetimes obtained from fitting to the sum of exponential decays, details shown in the Supporting Information.

The luminescence spectrum of **[7]Br** exhibits subtle vibrational features in toluene at ambient temperature, similar to **[7]H**.^[^ ^3^ ^]^ Namely, **[7]Br** displays fluorescence at a wavelength (*λ*_max,Fl_) of 447 nm accompanied by a noticeable shoulder at 411 nm. Furthermore, it exhibits phosphorescence at a wavelength (*λ*_max,Ph_) of 564 nm with a vibronic shoulder at 605 nm (Figure 2a); note that the literature for **[7]H** does not report any phosphorescence properties. Clearly, the integration of the heavy bromine atom into **[7]Br** plays a pivotal role in facilitating a pronounced and effective intersystem crossing (ISC) and the introduction of additional nonradiative triplet pathways results in a reduced photoluminescence quantum yield at room temperature for **[7]Br** (*Φ*_f_ = 0.8%) compared to **[7]H** (*Φ*_f_ = 2%). A notable difference is observed also in the excited‐state lifetimes between **[7]Br** and **[7]H**. For **[7]H**, a mono‐exponential decay with a lifetime of 19 ns is reported.^[^ ^3^ ^]^ In contrast, as shown by time‐correlated single photon counting experiments, the excited‐state dynamics of **[7]Br** is much faster with a multi‐exponential decay (Table 1 and Figure S1.10, Supporting Information). The fastest decay component exhibits a lifetime of 0.3 ns, contributing to 97.9% of the overall decay process. The second component, contributing to 1.8%, has a lifetime of 1.6 ns. The longest‐lived excited state, contributing to merely 0.3%, has a lifetime of 16 ns. This accelerated decay in **[7]Br** suggests that bromine incorporation leads to rapid nonradiative recombination of triplets that are effectively populated from the enhanced rate of ISC.

The UV–vis absorption and emission spectra of **TD[7]Br** in toluene at ambient conditions (298 K) and in 2‐MeTHF at 77 K are shown in Figure 2b. The UV–vis spectrum displays a pronounced absorption band (i.e., *ε* ≈ 44,750 m ^−1^ cm^−1^ at 313 nm) in the higher‐energy region, accompanied by a moderate absorption band between 350 and 400 nm (*ε* ≈ 12,770 m ^−1^ cm^−1^ at 366 nm and *ε* ≈ 11,550 m ^−1^ cm^−1^ at 385 nm). The incorporation of thiadiazole moieties into a [7]helicene structure results in a substantial increase in the overall molar extinction coefficients compared to their unsubstituted counterparts.^[^ ^3^ ^]^ The pronounced band below 330 nm is a characteristic of the *π*–π* transitions inherent to the helicenic structure. Consistent with prior findings,^[^ ^7^, ^8^ ^]^ the absorption band between 360 and 400 nm is, on the other hand, attributed to the charge‐transfer (CT)‐type transition of *π*‐electrons from the helicene to the thiadiazole acceptor group (see also theoretical analysis below). Remarkably, the molar absorption coefficients of **TD[7]Br** are comparable to those of **TD[7]H** ^[^ ^8^ ^]^ but are substantially greater than those of the [7]helicene with only one fused thiadiazole group.^[^ ^6^ ^]^

The emission band of **TD[7]Br**, similar to **TD[7]H**,^[^ ^8^ ^]^ exhibits a subtle structured nature in toluene at room temperature, which becomes more pronounced at 77 K (see Figure 2b). As noted earlier, the presence of the heavy bromine atom facilitates ISC, which in this case leads to efficient phosphorescence at 77 K, where the system is likely to have less energy than the reverse ISC (rISC) activation energy that would convert phosphorescent triplet states to fluorescent singlet states (vide infra). The *λ*_max,Fl_ value for **TD[7]Br** was identified at 451 nm and *λ*_max,Ph_ at 580 nm with a smaller peak at 614 nm; note that solvatochromism occurs in **TD[7]Br**, Figure S1.8 and S1.9, Supporting Information, arising from its donor–acceptor‐type molecular structure. Notably, **TD[7]Br** exhibits a *Φ*_f_ = 14%, which is a nearly 20‐fold increase compared to **TD[7]H**. Contrary to **TD[7]H** (for which a significantly reduced lifetime of 0.55 ns compared to **[7]H** was reported),^[^ ^3^, ^8^ ^]^ **TD[7]Br** decay is of bi‐exponential nature, with a rapid lifetime component at 0.4 ns (accounting for 90%) and a more extended lifetime component of 1.9 ns (representing 10%).

Finally, the corresponding absorption and emission spectra of **[6]Br** are shown in Figure 2c. The absorption spectrum reveals several distinct bands with a pronounced peak at 319 nm, exhibiting *ε* of 20,400 m ^−1^ cm^−1^, and smaller peaks at 391 nm (*ε* = 1040 m ^−1^ cm^−1^) and 415 nm (*ε* = 975 m ^−1^ cm^−1^) in the lower‐energy region. Nonbrominated carbo[6]helicene, **[6]H**, shows a weaker absorption band in chloroform at 350 nm (*ε *= 17,000 m ^−1^ cm^−11^) but exhibits a moderate absorption in the 300–350 nm range (*ε* ≈ 30,000 m ^−1^ cm^−1^) and a strong one at 270 nm (*ε* ≈ 50,000 m ^−1^ cm^−1^); see Figure S1.5, Supporting Information.

In toluene at room temperature, the fluorescence spectrum of **[6]Br** displays well‐structured features at 421 and 444 nm, with a shoulder at 473 nm, similar to **[6]H**, for which *λ*_max,Fl_ occurs at 420 nm with visible vibronic progression at 439 and 469 nm in chloroform (see Figure S1.6, Supporting Information). Note that here, again, the fluorescence quantum yield is found to be low *Φ*_f_ = 0.6%. Furthermore, for **[6]Br**, the phosphorescence peak wavelength *λ*_max,Ph_ at 77 K is detected at 528 nm with a clear vibronic substructure at 574 and 621 nm. Combined with low‐emission quantum yield, this enhanced phosphorescence indicates that bromination introduces more nonradiative recombination pathways from an efficiently populated triplet, owing to the bromine‐enhanced ISC.

It is worth noting that while the nonradiative rate constants are of the same order of magnitude for the three compounds, **[6]Br**, **[7]Br**, and **TD[7]Br** (≈2–5 × 10^9^ s^−1^, see Table 1), the latter displays a radiative rate constant that is one order of magnitude higher (3.5 × 10^8^ s^−1^ versus ≈3 × 10^7^ s^−1^). Overall, these results show the favorable combination of the bromine atom with the presence of the two thiadiazole units in the heptahelicenic core for the improvement of the emission process.

### 2.3 ECD Spectroscopy

While the photophysical properties influenced by the heavy‐atom effect have been extensively researched, chiroptical properties, such as ECD and CPL, remain rather underexplored in the literature.^[^ ^14^ ^]^

**Figure 3:**
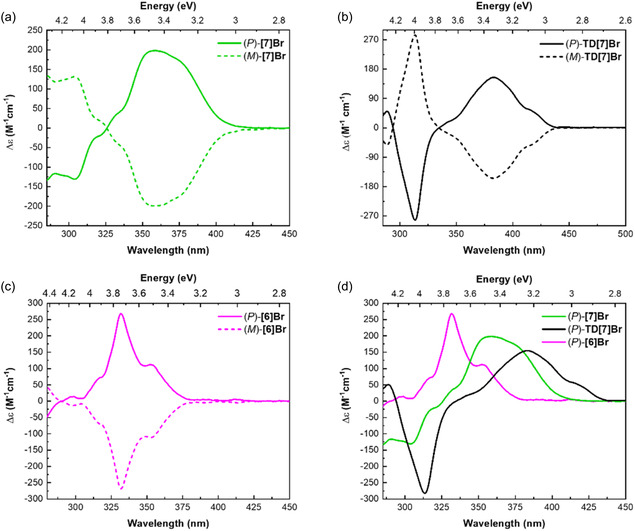
Mirror‐image ECD spectra of (*P*)‐ and (*M*)‐enantiomers of a) **[7]Br**, b) **TD[7]Br**, and c) **[6]Br** recorded in toluene at 298 K (*C* 1 × 10^−5^ m). d) A comparison of ECD spectra for (*P*)‐enantiomers of all three considered helicenes is presented.

The ECD spectra of enantiopure samples of **[7]Br**, **TD[7]H**, and **[6]Br** were measured under ambient conditions in toluene, as shown in **Figure** **3**, exhibiting the expected mirror‐image characteristics for the corresponding enantiomers and being consistent with conventional carbo[*n*]helicenes^[^ ^15^ ^]^ relationships between the helical chirality and the sign of the ECD signal the low‐energy region. The (*P*)‐isomer of **[7]Br** presents a positive ECD band at 357 nm with a molar ellipticity Δ*fε* = +200 m ^−1^ cm^−1^ and an additional, less pronounced shoulder at 380 nm (Δ*ε* = +156 m ^−1^ cm^−1^); see Figure 3a. In the higher‐energy region, a subtle shoulder at 330 nm (Δ*ε* = +31 m ^−1^ cm^−1^) is followed by an inversion in the ECD signal, exhibiting a weak shoulder at 321 nm (Δ*ε* = −20 m ^−1^ cm^−1^) and a more prominent negative peak at 304 nm (Δ*ε* = −132 m ^−1^ cm^−1^). Comparatively, as reported in the literature,^[^ ^3^ ^]^ the corresponding unsubstituted [7]helicene, **[7]H**, in its (*P*)‐enantiomeric form, displays a positive ECD band at 350 nm (Δ*ε* = +220 m ^−1^ cm^−1^) with a weaker shoulder at 380 nm (Δ*ε* = +180 m ^−1^ cm^−1^) and a low‐intensity one in the higher energies (330 nm, Δ*ε* = +40 m ^−1^ cm^−1^) that is followed by a reversal in sign, characterized by a notable peak at 305 nm (Δ*ε* = −100 m ^−1^ cm^−1^) and a more significant negative band at 275 nm (Δ*ε* = −450 m ^−1^ cm^−1^). Thus, despite the bromination, the overall shape of the ECD spectrum of **[7]Br** remains remarkably similar to that of its nonbrominated counterpart **[7]H**, particularly in the lower‐energy region. The higher‐energy part of the ECD spectrum of **[7]H** demonstrates greater anisotropy in absorption compared to **[7]Br**.

As illustrated in Figure 3b, the (*P*)‐enantiomer of **TD[7]Br** displays a positive Cotton effect at 383 nm (Δ*ε* = +159 m ^−1^ cm^−1^) along with a discernible shoulder at 416 nm (Δ*ε* = +54 m ^−1^ cm^−1^). In the higher‐energy range, a significant negative band was observed at 313 nm (Δ*ε* = −286 m ^−1^ cm^−1^), followed by another inversion in the sign. These spectral features bear a close resemblance to those of **TD[7]H** reported in the literature.^[^ ^8^ ^]^ Namely, for (*P*)‐**TD[7]H**, the lowest‐energy ECD signal was recorded with a Δ*ε* value of +55 m ^−1^ cm^−1^ at 420 nm, followed by a pronounced peak at 380 nm (Δ*ε* =+ 150 m ^−1^ cm^−1^). Upon sign reversal in the higher‐energy region, a notable peak was detected at 310 nm (Δ*ε* = −280 m ^−1^ cm^−1^), succeeded by another reversal in sign. This comparison highlights the similarities in the chiroptical properties of **TD[7]Br** and **TD[7]H**, particularly in their response to the Cotton effect across different energy domains.

Finally, the (*P*)‐**[6]Br** enantiomer shows a notable positive ECD band at 331 nm (Δ*ε* = +272 m ^−1^ cm^−1^) and a subsequent shoulder at 353 nm (Δ*ε* = +114 m ^−1^ cm^−1^), indicating strong ECD transitions (Figure 3c). Then, a shift toward negative Δ*ε* values is observed with a shoulder at 315 nm (Δ*ε* = +71 m ^−1^ cm^−1^), following negative intensity at higher energies. In comparison, (*P*)‐**[6]H** in chloroform displays a peak at 348 nm (Δ*ε* = +95 m ^−1^ cm^−1^), a strong band at 325 nm with Δ*ε* of +244 m ^−1^ cm^−1^, followed by a shoulder at 310 nm (Δ*ε* = +74 m ^−1^ cm^−1^) that changes into negative Δ*ε* region at higher energies, underscoring minimal differences induced by bromination (for the corresponding **[6]H** spectrum, see Figure S1.5, Supporting Information).

### 2.4 CPL Spectroscopy

**Figure 4:**
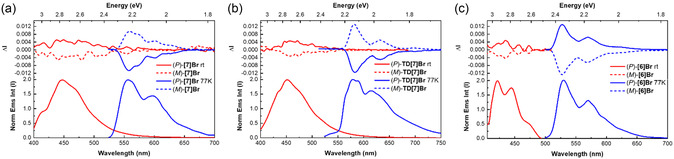
CPL (top) and normalized emission (bottom) spectra of a) **[7]Br**, b) **TD[7]Br**, and c) **[6]Br** in toluene at room temperature (rt) = 298 K (red line) and in 2‐MeTHF at 77 K (blue line), *C* 1 × 10^−5^ m. Spectra for (*P*)‐ and (*M*)‐isomers are represented by solid and dashed lines, respectively.

All three considered helicenes **[7]Br**, **TD[7]Br**, and **[6]Br** demonstrate CPL signals at both ambient temperature in toluene and at 77 K in 2‐MeTHF, mirror image for the (*P*)‐ and (*M*)‐enantiomers, as illustrated in **Figure** **4**. The CPL signals for **[7]Br** at room temperature correlate with the low‐energy ECD signals (Figure 4a); specifically, (*P*)‐**[7]Br** shows a positive CPL intensity, with measured *g*_lum_ value of +5 × 10^−3^ at 449 nm. At the cryogenic temperature of 77 K, a reversal in the CPL signal sign^[^ ^16^ ^]^ alongside a more pronounced vibrational progression and heightened chiroptical properties is visible. At this lower temperature, the *g*_lum_ values were observed to peak at −1.1 × 10^−2^ at 558 nm, indicating a significant enhancement in chiroptical behavior. It is also evident that the chiroptical responses from the singlet excited‐state *S*_1_ are opposite in sign to that of the triplet excited‐state *T*_1_.

As shown in Figure 4b, **TD[7]Br** and **[7]Br** exhibit similar trends in CPL responses. Namely, at room temperature, a sign of the CPL signal aligns with that of ECD, exemplified by the (*P*)‐**TD[7]Br**, which has a positive CPL intensity characterized by a *g*_lum_ value of +4 × 10^−3^ at 462 nm, while at 77 K a sign inversion occurs, again accompanied by a more evident vibrational progression and enhanced chiroptical activity. The *g*_lum_ values at 77 K reached −1.2 × 10^−2^ at 580 nm, marking one of the highest values reported to date for [7]helicenes.

Regarding **[6]Br**, as depicted in Figure 4c, there is, again, a notable correlation between the CPL and low‐energy ECD responses; (*P*)‐**[6]Br** displays a positive CPL signal with the recorded *g*_lum_ value of +6.5 × 10^−3^ at 434 nm. Interestingly, upon double bromination, the CPL seems to increase significantly, as *g*_lum_ value of **[6]H** is +1 × 10^−3^ at 420 nm in chloroform (see Figure S1.6, Supporting Information). Moreover, while upon cooling to 77 K, again, evident vibrational progression and an enhancement in chiroptical properties occur, the sign of CPL signal remains the same. At this reduced temperature, the *g*_lum_ values for (*P*)‐**[6]Br** reached a peak of 1.3 × 10^−2^ at 528 nm, demonstrating a significant alteration from [7]helicene bromides **[7]Br** and **TD[7]Br**.

**Figure 5:**
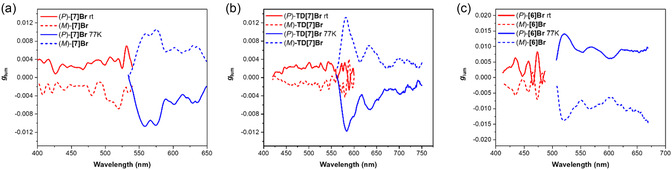
*g*_lum_ spectra of a) **[7]Br**, b) **TD[7]Br**, and c) **[6]Br** in toluene at room temperature (rt) = 298 K (red line) and in 2‐MeTHF at 77 K (blue line), *C* 1 × 10^−5^ m. Spectra for (*P*)‐ and (*M*)‐isomers are represented by solid and dashed lines, respectively.

It is crucial to recognize that the shape of the *g*_lum_ graph for both **[7]Br** and **TD[7]Br** helicenes closely mirrors the corresponding luminescence spectrum at 77 K (see **Figure** **5**a,b), likely due to the dominance of the *T*_1_ excited state. The vibronic progression of *T*_1_ induces some minor variations in the *g*_lum_ values; however, the overall shape of the *g*_lum_ spectrum remains strikingly similar to that of the luminescence spectra over the entire energy regime. This observation strongly suggests that when a single excited state predominantly governs the system, there is minimal variation in the chiroptical responses. At room temperature, however, the scenario becomes more intricate, as shown in Figure 5a,b. The observed bi‐exponential decay in the luminescence of **TD[7]Br** and tri‐exponential decay of **[7]Br** (vide supra) imply the involvement of multiple electronic excited states, along with their vibronic substates, contributing to the radiative processes. This complexity gives rise to variable *g*_lum_ values, each reflecting the combination of chiroptical responses of the different excited states. The diversity in energy levels of these states translates into varied chiroptical behaviors across different segments of the CPL spectrum. Therefore, the variation in *g*_lum_ values at room temperature is a direct manifestation of the complex interplay between multiple excited states and their distinct chiroptical responses in CPL spectroscopy. It is also noteworthy that for **[7]Br** at room temperature, one excited state dominates, contributing 97.8% to the observed phenomenon. In contrast, for **TD[7]Br**, the dominant excited state contributes 90% with the remaining 10% arising from a minor excited state. This observation highlights the nuanced and more sensitive nature of chiroptical properties under different excited state conditions and the significant impact of even minor excited states on the overall chiroptical behavior in CPL.

Interestingly, in contrast to [7]helicenes, the *g*_lum_ graph for **[6]Br** shows a clear structured shape at room temperature, see Figure 5c.^[^ ^17^ ^]^ At 298 K, the radiative decay in **[6]Br** is primarily governed (98.9%) by a single excited state, leading to a *g*_lum_ plot that closely mirrors the emission spectrum. However, at 77 K, the vibronic substructure is quite evident. We can clearly see vibronic progression of the *T*_1_ excited state inducing minor variations in the *g*_lum_ values. Consistent with the observations for **[7]Br** and **TD[7]Br**, the overall shape of the *g*_lum_ spectrum remains strikingly similar to that of the luminescence spectrum over the entire energy regime at 77 K. The observed anisotropy for the *T*_1_ state remains quite higher compared to the singlet excited state. However, unlike for the examined [7]helicene bromides, there is no sign inversion of *g*_lum_ signals for **[6]Br**, underscoring a distinct difference in its chiroptical behavior at cryogenic temperatures.

### 2.5 Theoretical Analysis

Quantum‐chemical studies on photophysical and chiroptical properties of all three considered brominated helicenes were then performed. A full set of calculated data along with a complete description of the employed computational details can be found in the Supporting Information.

**Figure 6:**
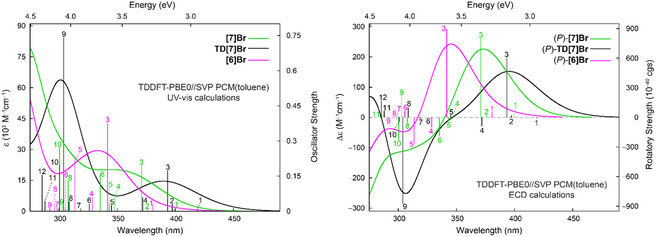
Simulated (TDDFT‐PBE0//SVP with continuum solvent model for toluene) UV–vis (left) and ECD (right) spectra for the considered brominated helicenes. No spectral shift has been applied. Selected calculated excitation energies along with the corresponding oscillator and rotatory strengths analyzed in detail (see the Supporting Information), indicated as “stick” spectra.

**Figure 7:**
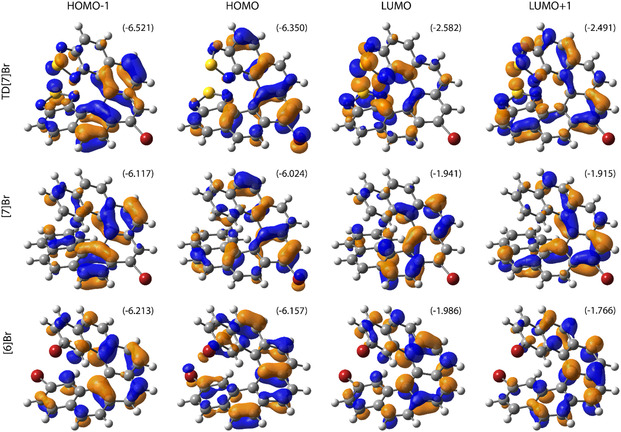
Comparison of isosurfaces (±0.04 au) of frontier MOs for the considered brominated helicenes. Based on PBE0//SVP with continuum solvent model for toluene calculations. In parentheses, orbital energies (in eV) are listed.

The resulting simulated time‐dependent density functional theory (TDDFT),^[^ ^18^ ^]^ employing the hybrid Perdew, Burke and Ernzerhof PBE0 functional, split‐valence polarization (SVP) basis set, and continuum solvent (toluene) model, UV–vis absorption and ECD spectra are presented in **Figure** **6**. Considering that no vibronic contribution has been taken into account, the calculations agree fairly well with the experiments as they overall correctly reproduce the extent of the lowest‐energy UV–vis absorption along with the energetic position of the low‐energy (positive for (*P*)‐enantiomers) and the sign patterns of ECD bands for the studied systems (see Figures 2 and 3). In particular, the experimental order of energy maxima of the aforementioned lowest‐energy ECD band across the series is correctly reproduced: **TD[7]Br** (lowest‐energy = longest‐wavelength) < **[7]Br** < **[6]Br** (highest‐energy = shortest wavelength). An analysis of underlying excitations (for the corresponding tables and figures, see the Supporting Information) demonstrates that this lower‐energy intensity in the UV–vis and ECD spectra for each of the systems stems predominantly from excitation no. 3, showing sizable oscillator and rotatory strength values and involving the frontier molecular orbitals (MOs): HOMO‐1, (HOMO) highest occupied molecular orbital, (LUMO) lowest unoccupied molecular orbital, and LUMO + 1 (see **Figure** **7**). Note that the lowest‐energy excitation no. 1, which originates mainly from the HOMO → LUMO electronic transition, affords in our calculations overall rather negligible intensity both in UV–vis absorption and in ECD spectra.

For the brominated [6]‐helicene **[6]Br**, the excitation no. 3 involves HOMO‐1 → LUMO + 1 (72%) and HOMO → LUMO (25%) transitions of predominantly *π*–π* character. Indeed, as shown in Figure 7, all these MOs correspond to an almost evenly spread out *π*‐electron system. Regarding **[7]Br** and **TD[7]Br**, this dominant excitation originates from HOMO → LUMO + 1 (53% and 60%, respectively) and HOMO‐1 → LUMO (39% and 37%) transitions. In the case of **[7]Br**, as for **[6]Br**, they are π–π* transitions localized predominantly in the central ([5]helicene) part of the molecule with some involvement of bromine orbital (see Figure 7). The experimentally observed redshift of the spectra for **[7]Br** versus **[6]Br** can be, thus, rationalized as due to the effect of enlargement of *π*‐electron system via the introduction of the additional aromatic ring that resembles a typical behavior of regular carbohelicenes and helicene‐like systems.^[^ ^15^, ^19^ ^]^ As expected, however, for **TD[7]Br**, due to the presence of strong electron‐withdrawing groups—thiadiazole moieties constituting terminal rings of the helical platform—a polarization of *π*‐electron density is visible in the frontier MOs with, for example, HOMO‐1 & HOMO/LUMO, predominantly localized on the *π*‐systems in the central part ([5]helicene, with Br in the case of HOMO)/the terminal rings (thiadiazole moieties) of the molecular structure (see Figure 7), inducing strong low‐energy helicene → thiadiazole CT contributions (particularly via HOMO‐1 → LUMO) to π–π* transitions. An inspection of the orbital energies also revealed that the introduction of thiadiazole rings in **TD[7]Br** instead of benzene in **[7]Br** has a noticeably stabilizing effect on the lower‐energy unoccupied levels, thus, reducing energetic gaps between higher‐energy occupied and lower‐energy unoccupied orbitals. All this may explain the most redshifted spectra observed for the compound.

**Figure 8:**
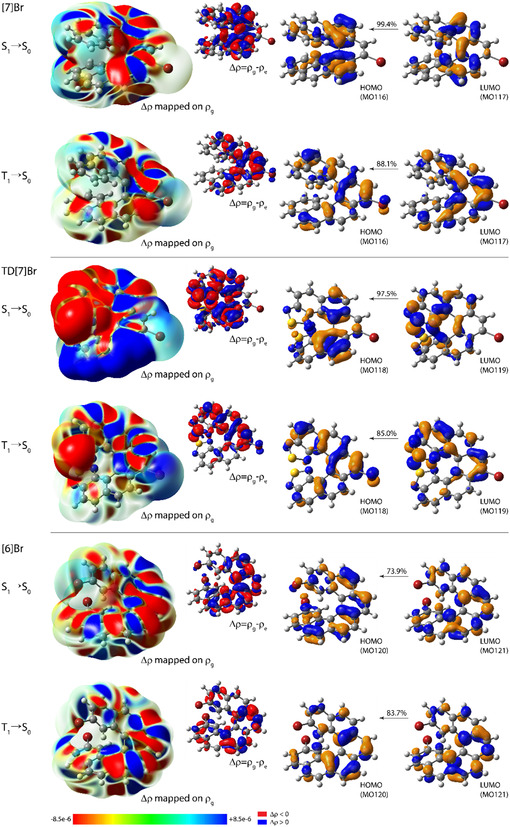
Electronic characteristics of *S*_1 _→ *S*_0_ fluorescence and *T*_1 _→ *S*_0_ phosphorescence (from, respectively, TDDFT–PBE0//SVP/PCM(toluene) and TDDFT–TDA–PBE0//SVP/PCM(THF) calculations) for (*P*)‐**[7]Br**, (*P*)‐**TD[7]Br**, and (*P*)‐**[6]Br**. Left: Isosurfaces (±0.0008 au) of the electron density differences between the *S*_0_ ground state and the respective excited state, Δ*ρ *= *ρ*_g_ − *ρ*_e_, and isosurfaces (0.0004 au) of *ρ*_g_ color mapped using the values of Δ*ρ*. Electron density moves from the red region to the blue region when moving from the excited state to the ground state. Right: The corresponding dominant MO‐pair contributions (isosurfaces: ±0.04 au) to emission transition.

To provide some insight into emission features observed experimentally across the examined series of compounds, the lowest‐energy singlet *S*_1_ and triplet *T*_1_ excited‐state geometry optimizations were performed, followed by excitation energy along with dipole (*D*) and rotatory strength (*R*) values calculations employing TDDFT (for *S*_1_) or TDDFT with Tamm–Dancoff approximation (TDDFT–TDA for *T*_1_) with SOC.^[^ ^20^ ^]^ The computed (TDDFT(–TDA)–PBE0//DZP with continuum solvent (toluene/THF for *S*_1_/*T*_1_) model and ZORA‐SOC at the corresponding TDDFT(–TDA)–PBE0//SVP‐optimized structures) results, including excitation energies, dipole, and rotatory strengths along with the corresponding luminescence dissymmetry factors defined as *g*_lum_ = 4*R*/*D,* are summarized in **Table** **2** (see also the Supporting Information), while in **Figure** **8** the electron density differences between the respective ground‐ and excited‐state visualized (based on the data obtained via TDDFT(–TDA)–PBE0//SVP with continuum solvent (toluene/THF for *S*_1_/*T*_1_) model geometry optimizations) for all the considered systems are presented along with the dominant MO‐pair contributions (in each case HOMO/LUMO). Note that negative (red)/positive (blue) values in the aforementioned electron density differences correspond to outflow/inflow of electron density, accompanying either *S*_1 _→ *S*_0_ fluorescence or *T*_1 _→ *S*_0_ phosphorescence transition.

**Table 2:** Experimental and calculated (TDDFT(–TDA)–PBE0//DZP with continuum solvent model and ZORA–SOC) emission data for the considered brominated helicenes ((*P*)‐enantiomers). See Figure 8 and the Supporting Information.

System	Calc.a)						Expt.b)	
		*E* (*λ*)	*D*	*R*	∠ (**d**, **m**)	*g*_lum_	*E* (*λ*)	*g*_lum_
**[7]Br**	*S*_1_	2.479 (500)	5.82 × 10^4^	4.64 × 10^2^	67	+3.19 × 10^−2^	2.774 (447)	+5 × 10^−3^
	*T*_1_	1.946 (637)	1.25 × 10^−1^	−5.37 × 10^−4^	117	−1.71 × 10^−2^	2.199 (564)	−1.1 × 10^−2^
**TD[7]Br**	*S*_1_	2.342 (529)	1.91 × 10^4^	2.52 × 10^2^	62	+5.28 × 10^−2^	2.749 (451)	+4 × 10^−3^
	*T*_1_	1.944 (638)	5.52 × 10^−2^	−5.25 × 10^−4^	116	−3.81 × 10^−2^	2.138 (580)	−1.2 × 10^−2^
**[6]Br**	*S*_1_	3.019 (411)	4.22 × 10^4^	9.27 × 10^1^	80	+8.78 × 10^−3^	2.945 (421)	+6.5 × 10^−3^
	*T*_1_	2.240 (554)	3.84 × 10^−2^	7.46 × 10^−6^	78	+7.78 × 10^−4^	2.344 (528)	+1.3 × 10^−2^

a) Calculated data: *E* (*λ*) − *S*_1_–*S*_0_ energy difference (in eV (in nm)) at TDDFT–PBE0//SVP/PCM(toluene)‐optimized *S*_1_ geometry (for *S*_1_) and *T*_1_–S_0_ energy difference (in eV (in nm)) at TDDFT–TDA–PBE0//SVP/PCM(THF)‐optimized *T*_1_ geometry (for *T*_1_), corresponding to respectively fluorescence and phosphorescence energy; *D = *3*f*/2*E* – dipole strength, in cgs units of 10^−40^ esu^2^ cm^2^, for *T*_1_: averaged over three triplet components; *R*, rotatory strength, in cgs units of 10^−40^ esu^2^ cm^2^, for *T*_1_: averaged over three triplet components; ∠ (**d**,**m**), angle between electric **d** and magnetic **m** transition dipole moments vectors, in degree, for *T*_1_: value for triplet component affording the highest‐magnitude *R*; *g*_lum_ = 4*R*/*D*, emission dissymmetry factor, for *T*_1_: based on averaged *R* and *D*;

b) Experimental data: *E* (*λ*) – emission maximum energy (in eV (in nm)); *g*_lum_ – emission dissymmetry factor. Fluorescence data from measurements in toluene at 298 K. Phosphorescence data from measurements in 2‐MeTHF at 77 K.

As shown in Table 2, the PBE0‐computed emission energies overall agree with the experimental values rather qualitatively than quantitatively, since in most cases (except for **[6]Br**) a visible (by ≈50–80 nm) overestimation of the wavelengths of experimental emission maxima can be observed; the differences between experimental fluorescence and phosphorescence maxima are nevertheless rather satisfactorily reproduced by calculations, for example, for **TD[7]Br**: 129 nm (expt.) versus 109 nm (calc.), Table 2. Similarly, a numerical agreement between measured and PBE0‐computed *g*_lum_ values can also be assessed as qualitative, as comparable orders of magnitude were obtained. The striking exception is **[6]Br**, for which the modeled *g*_lum,Ph_ is one order of magnitude lower than both experimental value and calculated results for the other compounds. This discrepancy can be attributed to deficiencies of the employed computational protocol/model, in particular, neglecting specific solvent effects and vibronic contributions. The latter indeed has been already recognized as important in shaping CPL in helicenic systems.^[^ ^21^ ^]^ Importantly, however, the performed SOC–TDDFT calculations correctly reproduce the sign of CPL signal associated with both fluorescence and phosphorescence observed experimentally for the examined compounds, namely, positive values of *g*_lum_ for fluorescence and phosphorescence of (*P*)‐enantiomer of [6]helicene **[6]Br**, along with positive/negative *g*_lum_ for fluorescence/phosphorescence of (*P*)‐enantiomers of [7]helicenes, **[7]Br** and **TD[7]Br**. Such a change in the sign of CPL originating from singlet and triplet excited states can be rationalized by a change of underlying electronic transition enforcing a change of the angle between electric (**d**)‐ and magnetic (**m**)‐transition dipole‐moments vectors; and thus, a sign of *R* (according to the equation *R* = |**d**| × |**m**| × cos(∠(**d**,**m**)) with an acute/obtuse angle translating into positive/negative *R*). Recollect that the luminescence dissymmetry factor is computationally defined as *g*_lum_ = 4*R*/*D*, and as the corresponding dipole strength is, by definition, positive, its sign is determined by the sign of the rotatory strength of the emission transition. Note that another reason for a change in a sign of *T*_1 _→ *S*_0_ versus *S*_1 _→ *S*_0_ CPL signal than the aforementioned alternation of character of the underlying excited state could be, for example, mixing of different (higher‐energy) electronic states into the emitting excited state,[^16^, ^22^] each bringing their own electric and magnetic transition dipole moments.

The analysis of electronic features of *S*_1 _→ *S*_0_ fluorescence and *T*_1 _→ *S*_0_ phosphorescence for the examined systems was, thus, performed, see Figure 8. As it is clearly demonstrated, both *S*_1 _→ *S*_0_ and *T*_1 _→ *S*_0_ emissions for **[6]Br**, while predominantly engaging HOMO with different *π*‐electron distribution, show essentially the same electronic characteristics corresponding to *π*–π* state (see alternating inflow (blue) and outflow (red) of electron density within the *π*‐system delocalized across almost the whole molecule, accompanying the transition from the excited state to the ground state visible in the electron density differences). This supports the similar (acute for (*P*)‐enantiomer) angles between **d** and **m** vectors (see Table 2 and the Supporting Information) and consequently the same sign of circularly polarized fluorescence and phosphorescence observed for the compound. Similarly, *π*–π* characteristics of *S*_1_ and *T*_1_ excited states can also be noticed for **[7]Br** but in this case, however, only the former remains symmetrically delocalized over the helicenic *π*‐framework (primarily its central part), whereas the latter appears localized mostly on one fragment of the molecule (upper in the figure) and involves the bromine atom. Analogous change in the spatial symmetry of *S*_1_ and *T*_1_ is visible also for **TD[7]Br**, for which additionally a clear CT contribution to an otherwise *π*–π* emission transition is present, represented by the shift of electron density from the thiadiazole ring(s) to the central part of the molecule in the case of *S*_1 _→ *S*_0_ (reflecting *S*_0 _→ *S*_1_ helicene → thiadiazole CT component, vide supra) and from the thiadiazole ring to the bromine atom in the case of *T*_1 _→ *S*_0_. The different spatial localization of the emitting singlet and triplet states in both **[7]Br** and **TD[7]Br**, accompanied by the different CT in *S*_1 _→ *S*_0_ and *T*_1 _→ *S*_0_ transitions in **TD[7]Br**, can account for the change of the angle between electric‐ and magnetic‐transition dipole‐moment vectors associated with the fluorescence and phosphorescence emission in these compounds (from acute to obtuse for (*P*)‐enantiomers, see Table 2 and the Supporting Information) and accordingly the sign of the corresponding CPL signals. This highlights an important role of both nonterminally grafted bromine atom and electron‐withdrawing thiadiazole moieties introduced onto helicenic platform in altering its chiroptical luminescence properties. Relations between the positioning of an ISC promoter, such as Br, and chromophore photophysical properties, have been indeed recognized in the literature.^[^ ^23^ ^]^ While our results appear to suggest that terminally, symmetrically introduced bromine atoms, like in **[6]Br**, ensure the preservation of the CPL sign between room‐temperature fluorescence and low‐temperature phosphorescence, further, more systematic studies are, however, required to offer a predictive framework for designing molecules with desired optical properties.

### 2.6 Transient Spectroscopy Studies

Time‐resolved photoluminescence (TRPL) laser‐based pump–probe studies are particularly useful for understanding the photophysics in bromohelicenes because they reveal electronic and/or structural relaxation processes in the excited state of such compounds. Indeed, thanks to their inherent helical structure, helicenes have already shown strong ISC and increased triplet yield. For this purpose, transient spectroscopies are becoming important techniques for the in‐depth studying of their excited states.^[^ ^24^ ^]^

**Figure 9:**
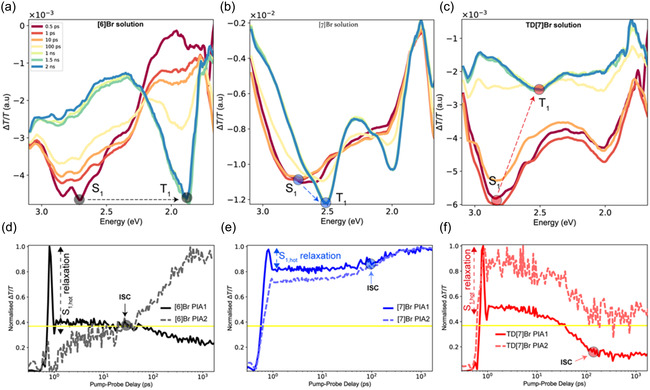
TA spectroscopy on the considered brominated helicenes. Spectral slices from 0.5 ps to 2.0 ns for a) **[6]Br**, b) **[7]Br**, and c) **TD[7]Br**. The legend in (a) applies to (b,c). In (a–c), the singlet state is labeled *S*_1_ and the triplet state is labeled *T*_1_. Kinetic traces of the labeled PIAs for the singlet and triplet states for d) **[6]Br**, e) **[7]Br**, and f) **TD[7]Br**. The *S*_1_ PIA is labeled PIA1 and *T*_1_ PIA is labeled PIA2 in all cases.

The transient absorption (TA) spectra recorded for all three considered brominated helicenes, **[7]Br**, **TD[7]Br**, and **[6]Br**, are shown in **Figure** **9**. The measurements were performed in degassed toluene solutions and at ambient temperature. We use a 400 nm pump, which precludes access to higher‐lying *S*_n_ states in the helicenes and thus, avoids internal conversion (IC) within a singlet excited‐state manifold. For all three brominated systems, we clearly observe ultrafast relaxation of the Frank–Condon state in the helicenes from a hot‐*S*_1_ excited state (either IC, or local excited (^1^LE)‐ to ^1^CT‐type transition). This stabilized *S*_1_ state now undergoes ISC populating triplet excited states. In the case of **[6]Br** (Figure 9a,d), we observe a singlet, *S*_1_, photoinduced absorption (PIA) with a peak at 2.7 eV. This evolves into a state that converts to another PIA feature with a peak at 1.9 eV; the latter is the slowly decaying emissive triplet excited state. Tracing the kinetics of these PIA features, we find that they crossover at 30 ps which is the characteristic time taken for ISC to occur. Notably, this is, however, much faster than the literature reports on the ISC process within helicenes^[^ ^24^ ^]^ and highlights the clear influence of the higher SOC afforded by the heavy bromine atoms. Overall, the fluorescence emission is not efficient because we see the triplet signal growing at the expense of the singlet. This is in agreement with the low fluorescence quantum yield found for **[6]Br**. The case of **[7]Br** (Figure 9b,e) shows ISC at 100 ps and small increase of the singlet and triplet photoinduced signals, which evolve similarly and slowly, suggesting a delayed kinetic in the singlet influenced by repopulation from the triplet manifold that we consider occurs through rISC (likely higher‐energy *T*_n_, taking into account relatively large *S*_1_/*T*_1_ energy gap determined via calculations (*vide supra*)). For **TD[7]Br** (Figure 9c,f), the emission is much more efficient with photoluminescence (PL) quantum yield (PLQY) of 14%. ISC also occurs at 100 ps, and the results suggest rISC‐mediated repopulation of the singlet (*vide infra*, Figure 11), which effectively slows the decay kinetics. The singlet and triplet PIA are hard to deconvolute in this case; however, the constant yet slow decay of the kinetics suggests that the rISC process leads to efficient recombination‐mediated decay in **TD[7]Br**, which gives higher PLQY.

**Figure 10:**
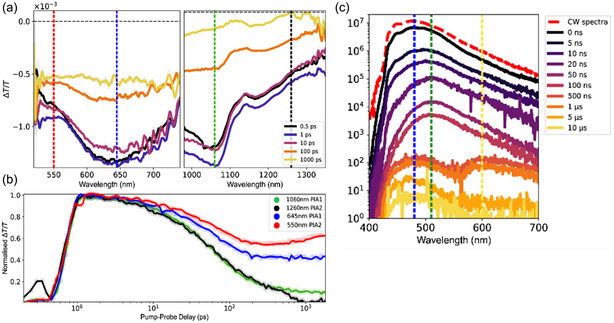
a) TA, b) time evolution of selected PIA signals, and c) transient emission spectra of **TD[7]Br** in degassed toluene at room temperature.

Due to the higher PLQY, we decided to examine **TD[7]Br** in detail using further TA and TRPL studies. After photoexcitation, ^1^LE state and ^1^CT state are formed. In **Figure** **10**a,b, the ^1^LE state has a PIA at 1260 nm band (black line) and has decayed by 90% within 1 ns (*τ* = 325.5 ps); since our emission lifetimes are in multiple nanoseconds, we can assign this state to be the first excited state that undergoes further CT to form the emissive ^1^CT state. The ^1^CT state can be attributed to the 2.25 eV PIA (550 nm) in Figure 10a and to the slower decaying PIA1 at 2.9 eV (427 nm) in Figure 10c, both of which grow within 1 ps and decay with lifetimes matching the majority PL decay. We observed earlier in Figure 9c how this ^1^CT PIA1 can undergo ISC/IC into the slower triplet state; given the parent singlet is of CT character, it is likely that this T state is also CT type and an alternative way to denote it would be ^3^CT.

TRPL studies are shown in Figure 10c. As visible, the first PL band is at 480 nm and it is likely to be photoluminescence from the ^1^CT state. We observe that this gives a way to second band at 510 nm, which decays slowly; this is the ^3^CT emission. A further lower‐energy PL evolves in μs timescales and is centered at 600 nm. This can be assigned to a lower‐lying triplet state, as it decays much more slowly than common radiative rates known for singlet states. This state is denoted as the ^3^CT_B_ state. It accounts for a very small population of the original exciton and most of PL is mediated by radiative decay of ^1^CT and ^3^CT states. Given the bi‐exponential behavior of the kinetics, it is likely that there is a thermally activated barrier for the ISC and rISC processes here. Through the time‐resolved spectroscopy, we are able to draw a possible picture of the exciton evolution, a fast CT from a singlet local exciton, ^1^LE, to a singlet CT‐type state, ^1^CT, within 1–2 ps is the first step, which is then followed by a rapid ISC to the ^3^CT state that is accelerated by the SOC afforded by a carefully placed bromine atom involved in the exciton. This ^3^CT state can radiatively and nonradiatively decay back to the ground state but as evidenced by TRPL, it can also convert to an even lower‐lying triplet CT state denoted as ^3^CT_B_.

In the analysis of CT states within the molecular framework, the second observed band pertains to the ^1^CT state, discernible in peaks assigned using both green and blue lines in the provided spectra (Figure 10c). This state can be directly excited or populated through energy transfer from the ^1^LE state, bridging specific donor‐like and acceptor‐like molecular fragments. The spectral overlap of the ^1^CT and ^1^LE bands leads to similar initial kinetics, yet the ^1^CT state diverges with a second kinetic component marked by a 3 ns lifetime, corresponding to the primary photoluminescence observed at 470 nm. Further, the ^3^CT state, identifiable by the red spectrum, exhibits an increase in population over time post 300 ps, suggesting this timeframe aligns with ISC under ambient conditions. Time‐resolved infrared TA reveals a bi‐exponential dynamic, with a 3 ns component and a possibly nonemissive component lasting 1.1 μs.

Regarding TRPL, three principal bands at 470, 510, and 600 nm are noted, with the first two contributing to nearly 99% of the total PL. These two bands correspond to the lifetimes of the ^1^CT and ^3^CT states, respectively. Notably, the 470 nm PL transitions smoothly to 510 nm within 50 ns, and an rISC process reinitiates PL at 470 nm. At longer timescales of 1–5 μs, a third PL band at 600 nm emerges, likely attributed to a slower, dark triplet level beneath the ^3^CT state.

**Figure 11:**
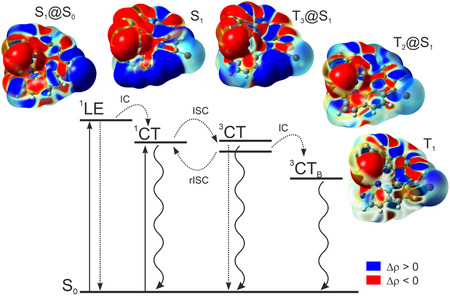
Jablonski diagram of proposed excited‐state dynamics for **TD[7]Br** with TDDFT(–TDA)‐postulated assignment of excited states. Solid/dotted arrow represents radiative/nonradiative process. Electron density transitions in individual excited states are shown as electron density differences between the *S*_0_ ground state and the respective excited state, Δ*ρ* = *ρ*_g_ − *ρ*_e_, color mapped on *ρ*_g_ (isosurfaces: 0.0004 au). Electron density moves from the red region (−8.5 × 10^−6^ au) to the blue region (8.5 × 10^−6^ au) when moving from the excited state to the ground state. For detailed characteristics of the presented states, see the Supporting Information.

While a detailed quantum‐chemical description of excited‐state processes requires applications of computationally expensive nonadiabatic molecular dynamics approaches and as such it surpasses the scope of this work, to shed some light on the proposed photophysics in **TD[7]Br**, the computed singlet–triplet energy gap and corresponding SOC strength values along with the electronic character of SOC‐coupled states were finally analyzed. The TDDFT–TDA–PBE0 calculations performed at the optimized *S*_1_‐**TD[7]Br** structure predict the presence of three triplet excited states of energy within close range to that of *S*_1_ (Δ*E*_S1/Tn_ = *E*_Tn_ − *E*_S1_: −0.52 eV for *T*_1_, −0.13 eV for *T*_2_, 0.02 eV for *T*_3_) and with the SOC interaction of ≈4–7 cm^−1^ (Table S2.7, Supporting Information), which appear to be typical values for phosphorescent organic helicene‐based compounds.[^24^, ^25^] Taking into account more favorable energy gaps, it could be tentatively suggested that the transition to the triplet manifold should mostly occur via *S*_1 _→ *T*_2_ and/or *S*_1 _→ *T*_3_ ISC, the latter driven not only by the lowest‐energy difference but also the highest SOC value obtained for the involved excited states, with all *S*_1_, *T*_2_, and *T*_3_, demonstrating (varying) CT character (see Table S2.8 and Figure S2.5, Supporting Information). As the SOC sizably mixes *T*_2_ & *T*_3_ with each other, with *T*_1_ and also with *S*_0_, after ISC, one can expect (apart from rISC to *S*_1_) either IC from these higher‐lying triplet states to lower‐lying *T*_1_ (subsequently emitting according to the Kasha's rule) or their radiative decay to the ground state. Note that the calculations indeed predict the emissive nature of these states (Table S2.8, Supporting Information). The excited‐state dynamics for the compound **TD[7]Br** proposed based on the experimental photophysical data and their discussion presented above and on the theoretical quantum‐chemical results can be accordingly schematically illustrated as in **Figure** **11** with ^1^CT assigned as *S*_1_, ^3^CT as either *T*_2_ or *T*_3_, and ^3^CT_B_ as *T*_1_.

## 3 Conclusion

In summary, we presented a comprehensive study on a series of three brominated helicenes, including the synthesis of a novel bromo‐heptahelicene‐bis‐thiadiazole derivative **TD[7]Br** and its characterization and detailed comparative photophysical and chiroptical analyses of these intriguing molecular systems. The research aimed to deepen the understanding of the dynamics of excited states and their influence on the chiroptical properties of helicenes that appear crucial for the development of novel CPL emitters. The introduction of bromine atoms was found to significantly affect the electronic properties of helicenes, facilitating the enhancement of ISC rates. As a result, **TD[7]Br** revealed a fluorescence quantum yield of 14% at room temperature. Furthermore, the comparison of **[7]Br** and **TD[7]Br** showed that the integration of thiadiazole not only stabilizes the triplet excited states but also modulates the emission properties, and exploiting processes, like rISC, lead to up to 20‐fold increment in emission quantum yield for **TD[7]Br**. The chiroptical investigations underscored the significant impact of molecular structure on the optical activity of these compounds, as illustrated by a phosphorescence dissymmetry factor of ±1.2 × 10^−2^ for **TD[7]Br** at low temperature. Complementing experimental findings, our theoretical studies using TDDFT provided deep insights into the electronic transitions and the nature of excited states of brominated helicenes. These computational models were instrumental in rationalizing the observed photophysical and chiroptical behaviors, offering some suggestions for designing molecules with desired optical properties^[^ ^26^ ^]^ for applications in advanced optoelectronic devices.

## Conflict of Interest

The authors declare no conflict of interest.

## Supporting information

Supplementary Material

## Data Availability

The data that support the findings of this study are available in the supplementary material of this article.
