# Supplementary material for: Bromo‐Heptahelicene‐Bis‐Thiadiazole: Photophysics, Chiroptics, and Excited‐State Dynamics
Source: Chemphyschem. 2025 May 12;26(15):e202500176. doi: 10.1002/cphc.202500176 (PMC12321275; doi:10.1002/cphc.202500176)
Supplement: Supplementary file 1 — Supplementary Material [file CPHC-26-e202500176-s001.pdf]

Supporting Information

Table of Contents

1. Experimental part S2

 1.1. General information S2

 1.2. Synthetic procedures S4

 1.3. NMR spectra S6

 1.4. Chiral HPLC separations S7

  1.4.1. Chiral separation of **TD[7]Br** S7

  1.4.2. Chiral separation of **[7]Br** S9

 1.5. X-ray crystallographic data S11

 1.6. Additional spectra S13

 1.7. References for experiments S17

2. Computational part S18

 2.1. Computational details S18

 2.2. Additional computed results S20

 2.3. Cartesian coordinates for optimized structures S32

 2.4. References for computations S35

1. Experimental part

1.1. General information

Materials and methods: Experiments were performed using standard Schlenk techniques. Column chromatography purifications were performed in air over silica gel (Macherey Nagel 60 M, 0.04–0.063 mm). Irradiation reactions were conducted using a Heraeus TQ 150 mercury vapor lamp. All reactions were monitored by TLC analysis, and visualizations were accomplished by irradiation with a UV light at 254 nm and at 356 nm. THF was dried using Na/Benzophenone method. Dry toluene was obtained from an MB-SPS-800 distillation machine and was degassed by an argon bubbling for at least 30 minutes before use. Other reagents and solvents were purchased from Sigma-Aldrich, Alfa Aesar, ABCR and Fluorochem, and were used as received unless otherwise noted.

NMR spectroscopy: ^{1}H and $^{13}C\{^{1}H\}$ NMR spectra were recorded at room temperature on Bruker Avance III 300 and 400 MHz spectrometers equipped with a tunable BBFO probe. Chemical shifts δ are given in ppm, and coupling constants J in Hz. ^{1}H and ^{13}C NMR chemical shifts were determined using residual signals of the deuterated solvents, either deuterated dichloromethane (^{1}H δ = 5.32 ppm, ^{13}C δ = 54.0 ppm) or deuterated chloroform (^{1}H δ = 7.26 ppm, ^{13}C δ = 77.2 ppm). The terms s, d, t, m indicate respectively singlet, doublet, triplet, multiplet, while dd stands for doublet of doublets. Assignments of proton and carbon signals are based on COSY, edited-HSQC, and HMBC experiments.

Chiral high-performance liquid chromatography (HPLC): HPLC was performed at Aix Marseille University, with Agilent Technologies 1260 Infinity unit. Chiralpak IE and Chiralpak IG are used as analytical (250 x 4.6 mm) and preparative (250 x 10 mm) columns.

High-resolution mass spectrometry (HRMS): HRMS measurements were performed at the CRMPO, University of Rennes 1, using electrospray ionization or matrix-assisted laser desorption/ionization (MALDI) as an ionization technique.

Polarimetry: Optical rotations were measured on a Jasco P-2000 polarimeter with a sodium lamp (589 nm) in a 3 cm cell, thermally equilibrated at 25 °C. Specific rotation parameters are given in $deg \cdot mL \cdot g^{-1} \cdot dm^{-1}$ (= 10^{-1} $deg \cdot cm^{2} \cdot g^{-1}$).

UV-vis spectroscopy and electronic circular dichroism (ECD): UV-vis spectroscopy measurements were conducted on a Jasco-V630 spectrometer. Electronic circular dichroism (in $M^{-1} \cdot cm^{-1}$) was measured on a Jasco J-815 Circular Dichroism Spectrometer IFR140 facility, using the PRISM core (Biogenouest©, UMS Biosit, Université de Rennes 1 - Rennes Cedex, France).

Luminescence spectroscopy: Solution state emission measurements were carried out in respective UV-grade solvent in quartz cuvettes of 1 cm path length. Spectra were recorded on a Jobin Yvon Fluoromax-2 spectrofluorimeter with an R928 PMT detector and corrected for the wavelength dependence of the monochromator and detector. Luminescence quantum yields

were determined using as the standard either quinine sulphate in an 0.5 M H_2SO_4 solution (φ = 0.546) or coumarin 153 in ethanol (φ = 0.40).[1.1] Luminescence lifetimes were measured in CAPHTER, University of Rennes 1. Lifetime measurements were realized using a picosecond laser diode (Jobin Yvon deltadiode, 375 nm) and a Hamamatsu C10910-25 streak camera mounted with a slow single sweep unit. Signals were integrated on the whole emission decay. Fits were calculated using the Hamamatsu software.

Circularly polarized luminescence (CPL): CPL measurements were performed using a home-built CPL spectrofluoropolarimeter (constructed with the help of the JASCO company). The samples were excited using a 90° geometry with a xenon ozone-free lamp 150 W LS. The following parameters were used: emission slit width ≈ 2 mm, integration time = 4 sec, scan speed = 50 nm/min.

Low-temperature CPL spectra for all the compounds investigated in this study were recorded in frozen 2-methyltetrahydrofuran (2-MeTHF) at 77 K. The measurements were conducted using a specialized optical cuvette equipped with a thermally insulated cryogenic flask designed to maintain liquid nitrogen temperature throughout the experiment. To ensure data integrity and avoid signal interference, meticulous precautions were taken to prevent condensation or ice formation both within and on the exterior surfaces of the cuvette. Excitation was performed using a 150 W ozone-free xenon lamp in a 90° geometry configuration. The instrument was operated under the following optimized parameters: emission slit width set to 2 mm, integration time of 4 seconds, five spectral accumulations, and a scan speed of 50 nm/min.

Transient photoluminescence (TRPL) spectroscopy: TRPL spectra at nanosecond-microsecond timescales were recorded using an electronically gated intensified CCD (ICCD) camera (Andor iStar DH740 CCI-010) connected to a calibrated grating spectrometer (Andor SR303i). A narrowband non-colinear optical parametric amplifier pumped with a frequency doubled output of a 1 kHz 800 nm laser pulse from a Ti:sapphire amplifier was used to generate a tunable 250-fs excitation pulse. Suitable long-pass filters (Edmund Optics) were used to prevent scattered laser signals from entering the spectrometer. Temporal evolution of the PL emission was obtained by stepping the ICCD delay with respect to the excitation pulse, with a minimum gate width of 5 ns. The raw data was corrected to account for filter transmission and camera sensitivity.

Transient absorption (TA) spectroscopy: TA experiments were conducted on a setup pumped by a regenerative Ti:sapphire amplifier (Solstice Ace, Spectra-Physics) emitting 100-fs pulses centred at 800 nm at a rate of 1 kHz and a total output of 7 W. Depending on the probed spectral range and timescales, different combinations of optical systems were used.

To collect sub-nanosecond dynamics in the visible range, frequency-doubled output of the amplifier was used to seed a home-built broadband non-collinear optical parametric amplifier (NOPA) tuned to output 540–750 nm pulses with a beta barium borate mixing crystal (Eksma Optics). Following chirp-correction the white light output was split on a 50/50 beam splitter,

focused to below 200 μm and used as the probe and reference beams. Wavelength-tunable pump pulses were generated in a home-built visible narrowband NOPA.

Alternatively, to probe the infrared range the output of the amplifier was used to seed a home-built NOPA tuned to output 1,250–1,700 nm pulses with a periodically poled stoichiometric lithium tantalate (PPLST) mixing crystal. The pump and probe beams were spatially overlapped at the focal point using a beam profiler, with the pump spot diameter at least fivefold larger than the probe. Time resolution was achieved by the introduction of a stepped optical delay (Thorlabs DDS300-E/M) between pump and probe pulses, with a computer-controlled delay stage allowing for maximum delay of 1.9 ns and beam wander of the probe due to changing beam pointing minimized to below 5 μm using a beam profiler. Pump pulses were chopped at 500 Hz to enable shot-to-shot referencing, which accounted for intensity fluctuations in the amplifier. After passing through the sample, the probe and reference beams were dispersed with a grating spectrometer (Shamrock SR303i, Andor Technology) and simultaneously measured with charge-coupled device (CCD) detector arrays (Entwicklungsbüro Stresing).

Finally, to collect sub-nanosecond dynamics in the ultraviolet range, the output of the amplifier was used to seed a home-built broadband NOPA tuned to output 350–650 nm pulses generated by focusing the 800 nm fundamental beam onto a CaF2 crystal (Eksma Optics, 5 mm) connected to a digital motion controller (Mercury C-863 DC Motor Controller), after passing through a mechanical delay stage. The transmitted pulses were collected with a single-line scan camera (JAI SW-4000M-PMCL) after passing through a spectrograph (Andor Shamrock SR-163).

1.2. Synthetic procedures

(2-Bromo-1,4-phenylene)bis(methylene) bis(triphenylphosphonium) dibromide **1** and racemic 9-bromo-heptahelicene **[7]Br** were prepared according to the literature.[1,2]

5,5'-((2-bromo-1,4-phenylene)bis(ethene-2,1-diyl))bis(benzo[c][1,2,5]thiadiazole) (3)

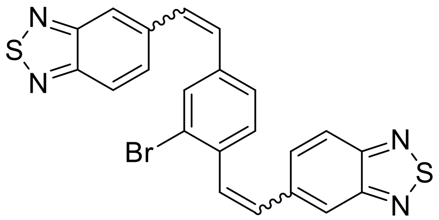

3

Within a flame-dried Schlenk flask outfitted with a magnetic stirring bar and sealed with a rubber septum, a 2.5 M solution of *n*-butyllithium (*n*-BuLi) in hexane (0.9 mL, 2.07 mmol, 2.07 eq.) was gradually introduced to a tetrahydrofuran (THF) solution (10 mL) of compound **1** (0.87 g, 1.00 mmol, 1.00 eq.) at -80 ºC under an argon atmosphere. The resulting mixture was stirred at this low temperature for 20 minutes. Subsequently, a THF solution (5 mL) of

2,1,3-benzothiadiazole-5-carbaldehyde **2** (0.35 g, 2.13 mmol, 2.13 eq.) was added dropwise to the aforementioned mixture at -80 °C under argon. Following this addition, the rubber septum was exchanged for a Teflon cap, and the reaction mixture was permitted to gradually return to ambient temperature over a period of 3 hours. Following the reaction, the solvent was removed under vacuum and the residue underwent purification via column chromatography on silica gel (SiO_2, using an *n*-heptane/EtOAc = 1:2 elution mixture) to yield 5,5'-((2-bromo-1,4-phenylene)bis(ethene-2,1-diyl))bis(benzo[c][1,2,5]thiadiazole) (compound **3**) as a yellow solid (0.19 g, 0.4 mmol, yield = 40%).

^{1}H NMR (300 MHz, CD_2Cl_2, *δ* ppm): Due to the *cis,cis*, *cis,trans* and *trans,trans* isomeric mixture, it was not possible to clearly assign proton signals (see ^{1}H NMR spectrum in Figure S1.1).

HRMS (ESI): m/z calculated for $[M+H^+]$ $[C_{22}H_{14}N_4{}^{79}BrS_2]^+$: 476.9838, observed 476.9836.

Bromo-[7]-Helicene Bis-thiadiazole (TD[7]Br)

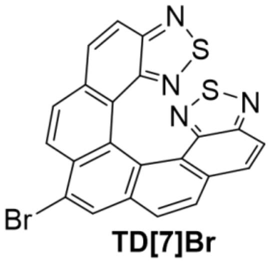

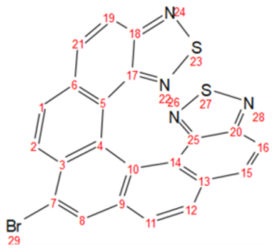

Precursor **3** in an isomeric mixture (0.10 g, 0.21 mmol, 1.00 eq.) was dissolved in toluene (2 L) and stoichiometric amounts of iodine (0.11 g, 0.42 mmol, 2.00 eq.) and an excess of propylene oxide (0.32 g, 4.20 mmol, 20.00 eq.) were added. The mixture was bubbled with argon for 30 minutes and subsequently irradiated for 14 hours using a Heraeus TQ 150 mercury vapor lamp. After evaporation of toluene, the crude was dissolved in ethyl acetate (40 mL) and washed three times with saturated solution of sodium thiosulphate (3 x 50 mL). The organic layer was dried over magnesium sulphate and concentrated by evaporation. The recovered solid was washed three times with diethyl ether (3 x 50 mL) to afford pure product **TD[7]Br** as a yellow solid (0.09 g, 0.19 mmol, yield = 90%).

^{1}H NMR (300 MHz, CD_2Cl_2, *δ* ppm): 7.85-7.90 (dd, J = 9.1 Hz, J = 8.4 Hz, 2H, H_{16}+H_{19}), 8.08-8.24 (m, 5H, H_1+H_{11}+H_{12}+H_{15}+H_{21}), 8.40 (s, 1H, H_8), 8.67-8.64 (d, J = 8.6 Hz, 1H, H_2).

^{13}C{^{1}H} NMR (101 MHz, CD_2Cl_2, *δ* ppm): 120.0 (s, CH, C_{16}), 120.4 (s, CH, C_{19}), 122.4 (s, C_7), 125.6 (s, C_9), 126.3 (s, C_6), 126.5 (s, C_{13}), 127.7 (s, C_5), 127.8 (s, CH, C_2), 128.0 (s, C_{12}), 129.4 (s, CH, C_1), 129.4 (s, CH, C_{11}), 131.4 (s, CH, C_8), 132.0 (s, C_{10}), 132.2 (s, C_3), 132.2 (s, C_4), 133.1 (s, C_{15}), 133.3 (s, CH, C_{21}), 133.9 (s, C_{14}), 153.0 (s, C_{20}-N), 153.1 (s, C_{18}-N), 154.4 (s, C_{17}-N), 154.4 (s, C_{25}-N).

HRMS (ESI): m/z calculated for $[M]^+$ $[C_{22}H_9N_4{}^{79}BrS_2]^+$: 471.9446; found 471.9446.

1.3. NMR spectra

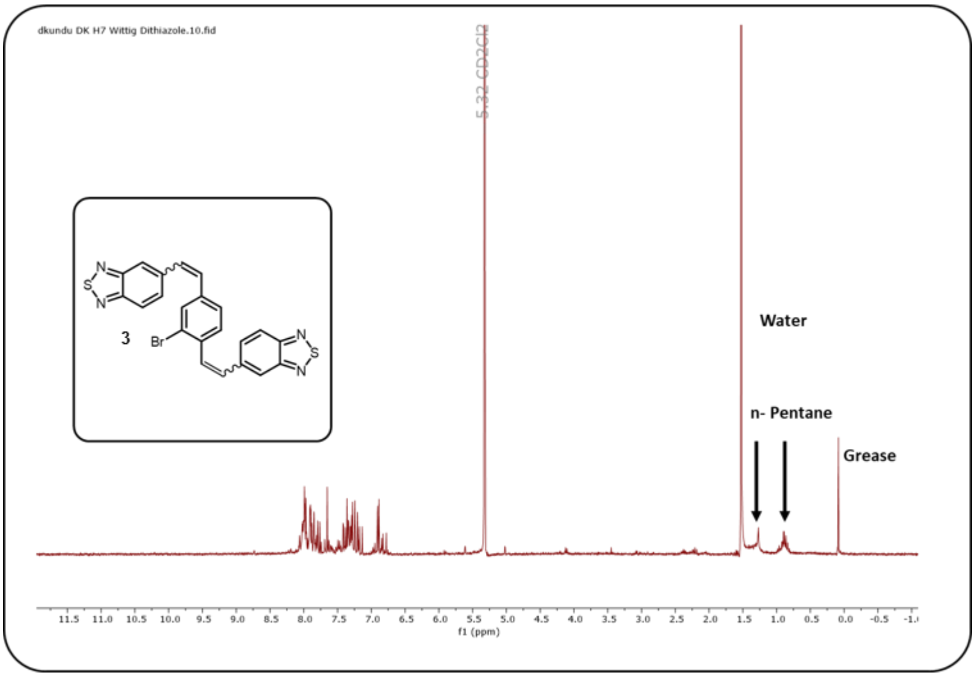

Figure S1.1: ^{1}H NMR spectrum of **3** in CD_2Cl_2 at 298 K (300 MHz).

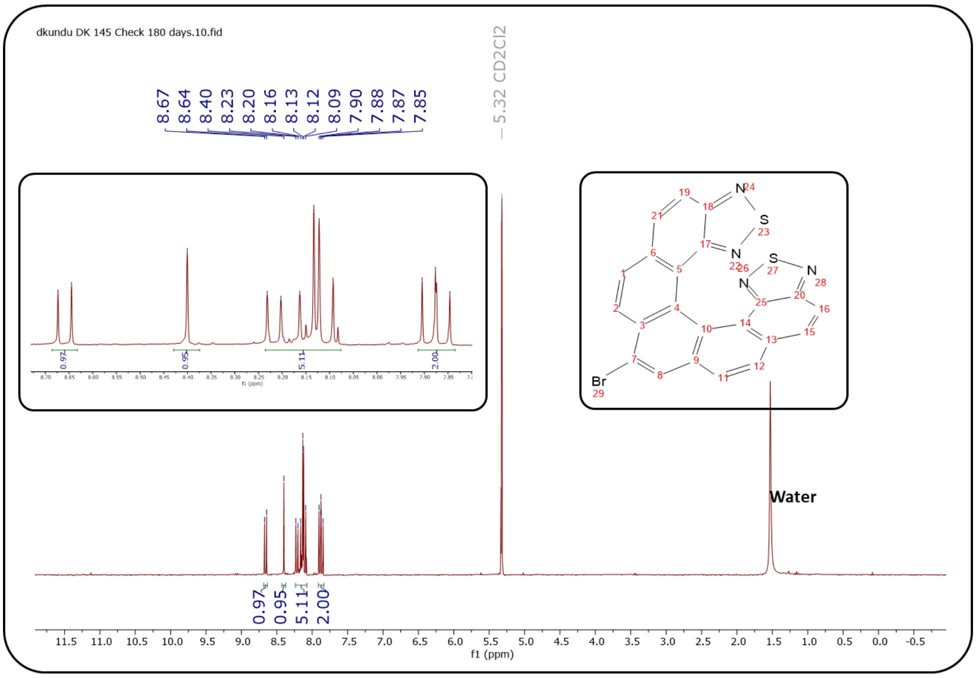

Figure S1.2: ^{1}H NMR spectrum of **TD[7]Br** in CD_2Cl_2 at 298 K (300 MHz).

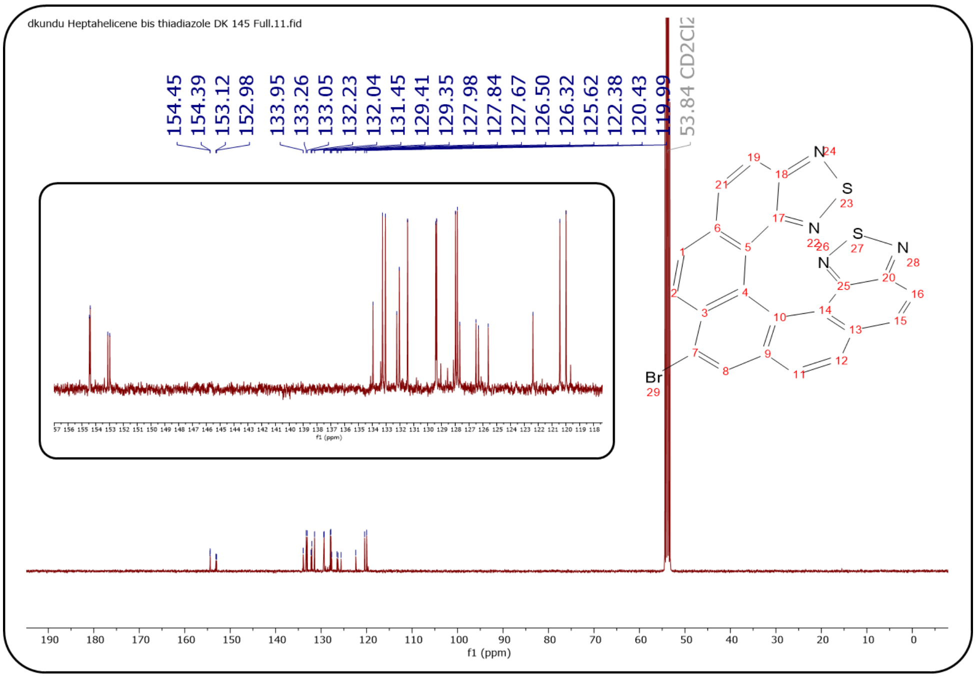

Figure S1.3: $^{13}C\{^{1}H\}$ NMR spectrum of **TD[7]Br** in CD_2Cl_2 at 298 K (101 MHz).

1.4. Chiral HPLC separations

1.4.1. Chiral separation of **TD[7]Br**

1.4.1.1. Analytical chiral HPLC separation for compound **TD[7]Br**

• The sample is dissolved in dichloromethane, injected on the chiral column, and detected with a UV detector at 230 nm. The flow-rate is 1 mL/min.

Column	Mobile Phase	t1	k1	t2	k2	α	Rs
**Chiralpak IG**	heptane / ethanol / dichloromethane (50/30/20)	4.71	0.60	5.58	0.89	1.49	3.74

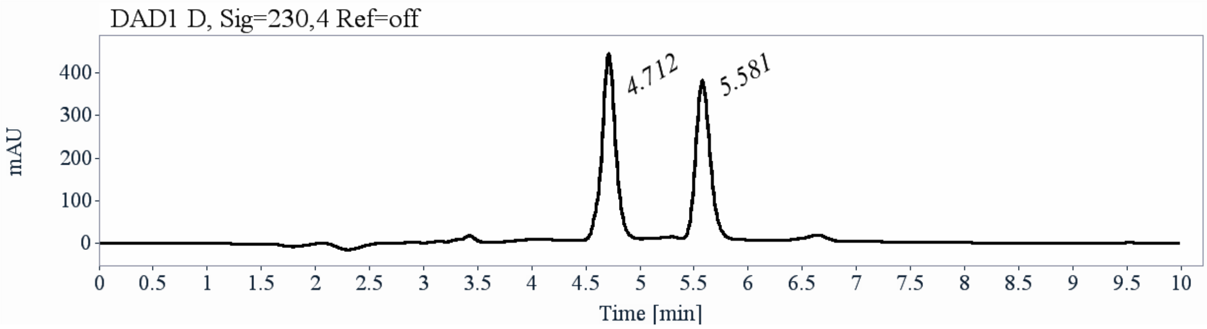

RT [min]	Area	Area%	Capacity Factor	Enantioselectivity	Resolution (USP)
4.71	3824	52.69	0.60	–	–
5.58	3434	47.31	0.89	1.49	3.74
Sum	7258	100.00	–	–	–

1.4.1.2. Preparative separation for compound **TD[7]Br**

• Sample preparation: About 40 mg of compound **TD[7]Br** are dissolved in 17 mL of dichloromethane.

• Chromatographic conditions: Chiralpak IG (250 x 10 mm), hexane / ethanol / dichloromethane (50/30/20) as mobile phase, flow-rate = 5 mL/min, UV detection at 254 nm.

• Injections (stacked): 85 times 200 µL, every 4 minutes.

• First fraction: 9 mg of the first eluted with *ee* > 99.5%

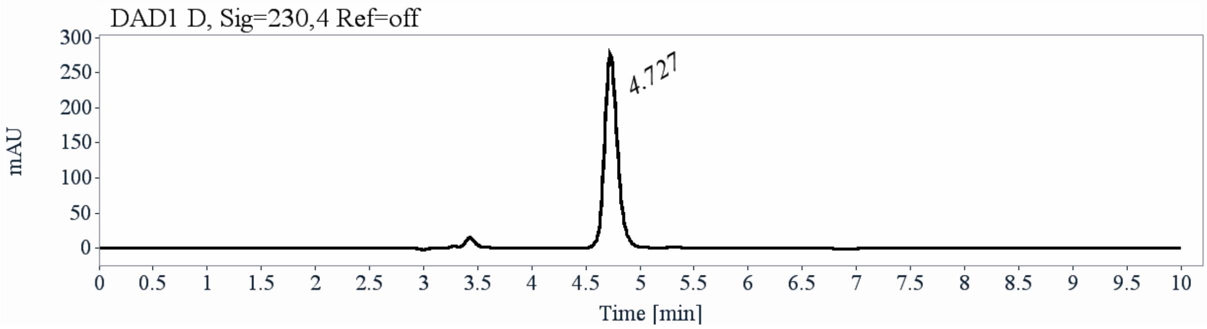

RT [min]	Area	Area%
4.73	2295	100.00
Sum	2295	100.00

• Second fraction: 11 mg of the second eluted with *ee* > 99.5%

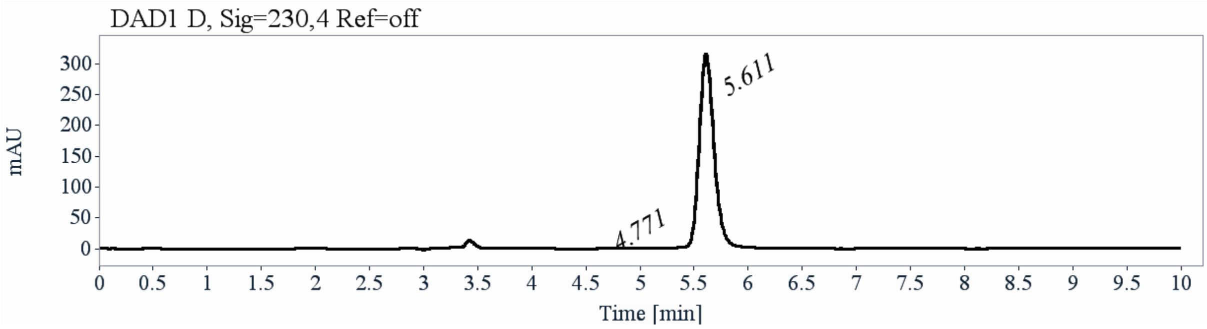

RT [min]	Area	Area%
4.77	6	0.20
5.61	2998	99.80
Sum	3004	100.00

1.4.2. Chiral separation of **[7]Br**

1.4.2.1. Analytical chiral HPLC separation for compound **[7]Br**

• The sample is dissolved in dichloromethane, injected on the chiral column, and detected with a UV detector at 254 nm. The flow-rate is 1 mL/min.

Column	Mobile Phase	t1	k1	t2	k2	α	Rs
**Chiralpak IE**	heptane / dichloromethane (70/30)	5.13	0.74	7.18	1.43	1.94	7.88

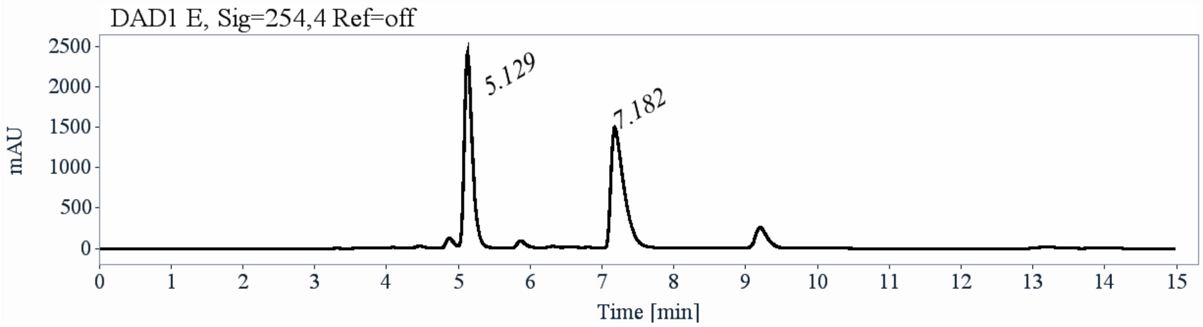

RT [min]	Area	Area%	Capacity Factor	Enantioselectivity	Resolution (USP)
5.13	18233	49.01	0.74	–	–
7.18	18968	50.99	1.43	1.94	7.88
Sum	37200	100.00	–	–	–

1.4.2.2. Preparative chiral HPLC separation for compound **[7]Br**

• Sample preparation: About 60 mg of compound **[7]Br** are dissolved in 6 mL of a mixture of dichloromethane and hexane (50/50).

• Chromatographic conditions: Chiralpak IE (250 x 10 mm), hexane / dichloromethane (70/30) as mobile phase, flow-rate = 5 mL/min, UV detection at 320 nm.

• Injections (stacked): 30 times 200 µL, every 8 minutes.

• First fraction: 22 mg of the first eluted with *ee* > 99.5%

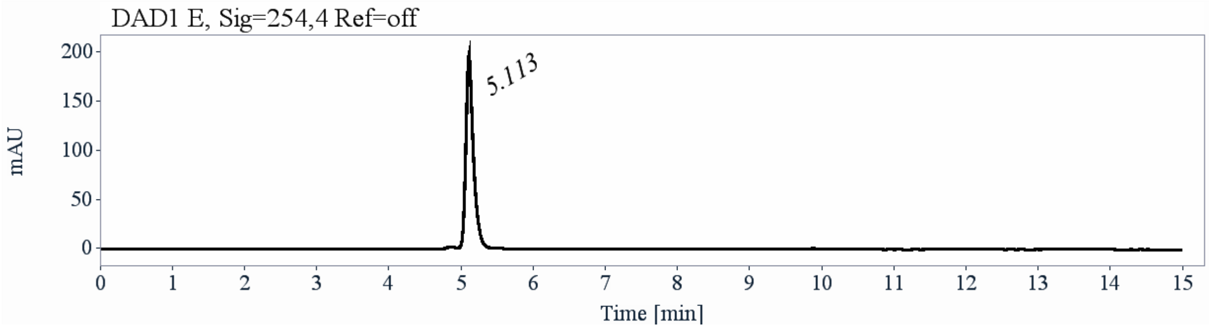

RT [min]	Area	Area%
5.11	1443	100.00
Sum	1443	100.00

• Second fraction: 22 mg of the second eluted with *ee* > 99.5%

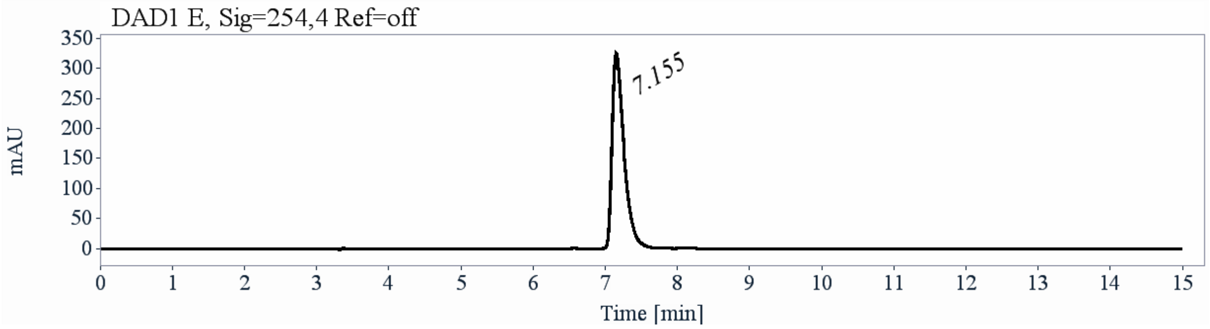

RT [min]	Area	Area%
7.16	3639	100.00
Sum	3639	100.00

1.5. X-ray crystallographic data

The data were collected at low temperature (150 K) on a D8 Venture Bruker-AXS diffractometer equipped with a CMOS-PHOTON70 detector, using MoKα radiation (λ = 0.71073 Å). The structure was solved using SHELXT 2018_2. H-atom parameters were constrained. All non-hydrogen atoms were refined with full-matrix least-squares on F^2 SHELXL-2018.

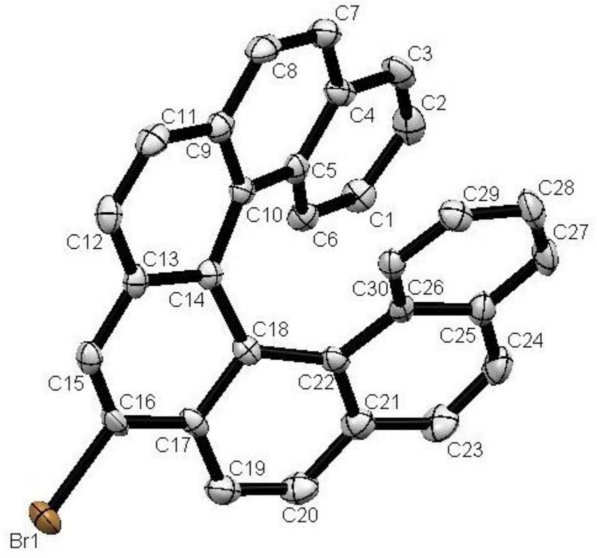

Figure S1.4: ORTEP figure (thermal ellipsoids represent 50% probability) from X-ray diffraction experiment for **[7]Br**. Hydrogen atoms are omitted for clarity.

Table S1.1: X-ray crystallographic data for (*rac*)-**[7]Br**.

**Empirical Formula**	$C_{30}H_{17}Br$	**$d_{calculated}$ ($g{\cdot}cm^{-3}$)**	1.527
**CCDC number**	2424610	**Absorption coefficient (mm^{-1})**	2.083
**Formula weight**	457.35	**Tmin**	0.541
**Temperature (K)**	150	**Tmax**	0.779
**Wavelenght (Å)**	0.71073	**F (000)**	464.0
**Crystal system**	Triclinic	**Crystal size (mm)**	0.450 x 0.130 x 0.120
**Space group**	*P*-1	**Θ range for data collection (°)**	2.556 to 27.521
**a (Å)**	8.0478(12)	**Limiting indices**	$-10 \leq h \leq 10$; $-14 \leq k \leq 14$; $-14 \leq l \leq 14$
**b (Å)**	11.1741(17)	**Data completeness**	98.0%
**c (Å)**	11.1934(16)	**Reflection collected**	4502
**α (°)**	88.380(5)	**Reflections uniques**	4502 [R(int) = complete]
**β (°)**	89.679(5)	**Data / restraints / parameters**	4502 / 0 / 281
**γ (°)**	81.207(5)	**Goodness-on-fit on F^2**	1.115
**Volume ($Å^3$)**	994.4(3)	**Final *R* indices [$I > 2\sigma$]**	R1 = 0.0774, wR2 = 0.2029
**Z**	2	**R indices (all data)**	R1 = 0.0905, wR2 = 0.2096
**Color**	Yellow	**Largest diff peak and hole ($eÅ^{-3}$)**	1.332 and -0.866

1.6. Additional spectra

The specific and molar rotations of the enantiopure helicenes were measured with a polarimeter in toluene at 298 K. Within an experimental error range of ±5%, the data show quite similar magnitudes. Obtained values are listed in Table S1.2.

Table S1.2: Specific and molar optical rotations of considered [7]-helicene bromides.

Compound	$[\alpha]_{589}^{25}$ (10^{-1} deg·cm^2·g^{-1})	$[\varphi]_{589}^{25}$ (10^{-1} deg·cm^2·mol^{-1})
(*P*)-**[7]Br**	+2380	+10,800
(*M*)-**[7]Br**	–2350	–10,700
(*P*)-**TD[7]Br**	+5980	+28,300
(*M*)-**TD[7]Br**	–6150	–29,100

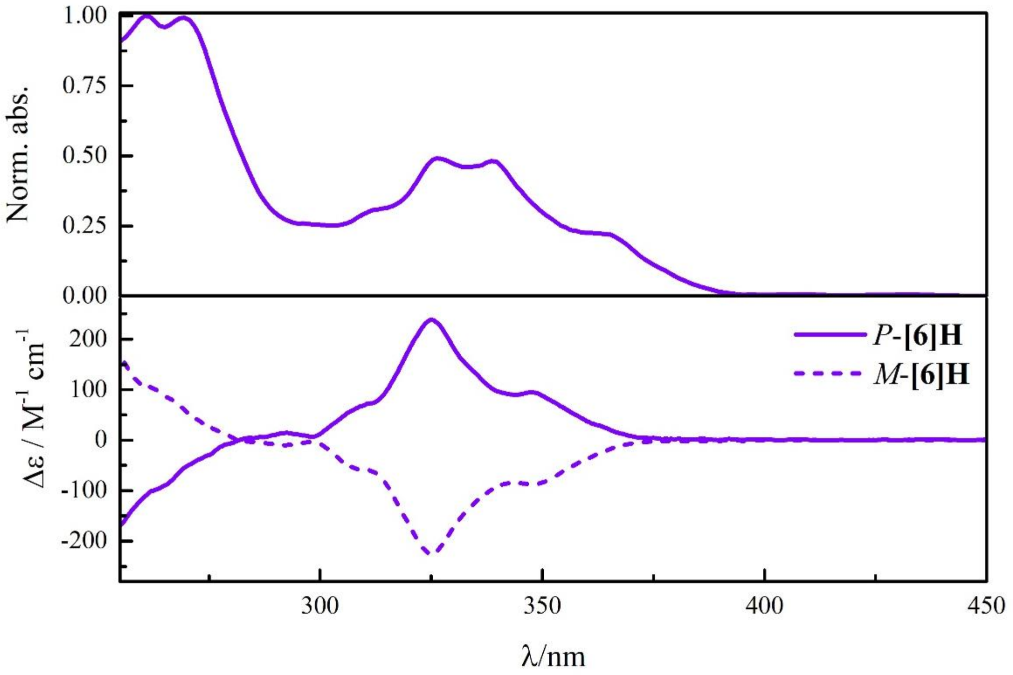

Figure S1.5: UV-vis and ECD spectra of **[6]H** (dark purple line) in chloroform at 298 K (C 1 × 10^{-5} M). Spectra for (*P*)- and (*M*)-isomers of [6]-helicene are represented by solid and dashed lines, respectively.

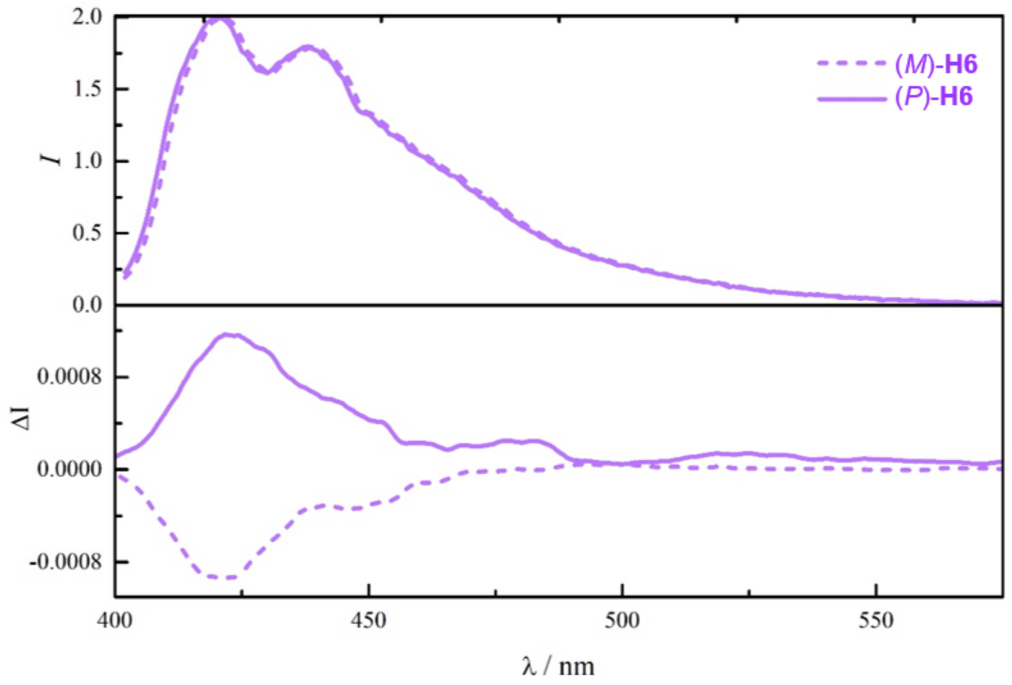

Figure S1.6: Emission (top) and CPL (bottom) spectra of **[6]H** in chloroform at 298 K (purple line) (C 1 × 10^{-5} M). Spectra for (P)- and (M)-isomers of **[6]H** are represented by solid and dashed lines, respectively.

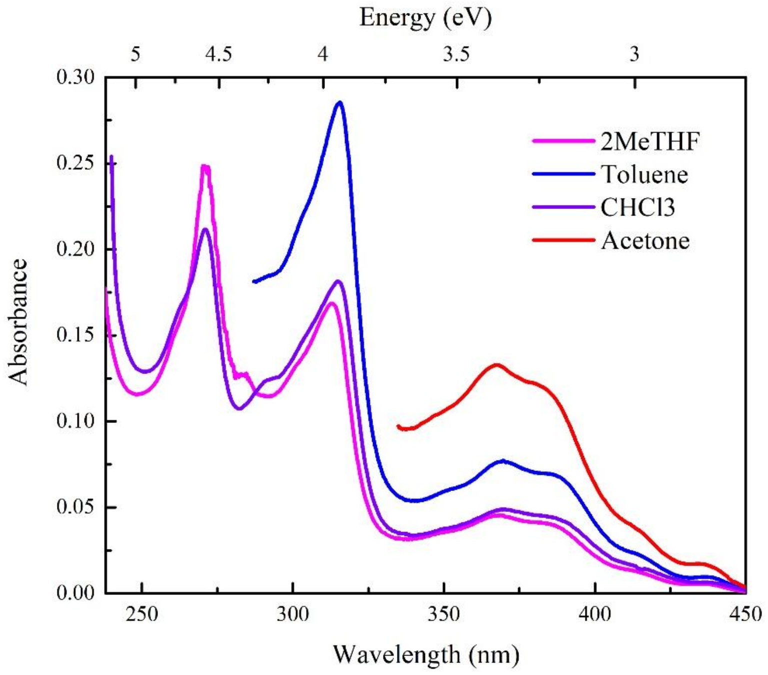

Figure S1.7: Absorption spectrum of **TD[7]Br** in different solvents at 298 K.

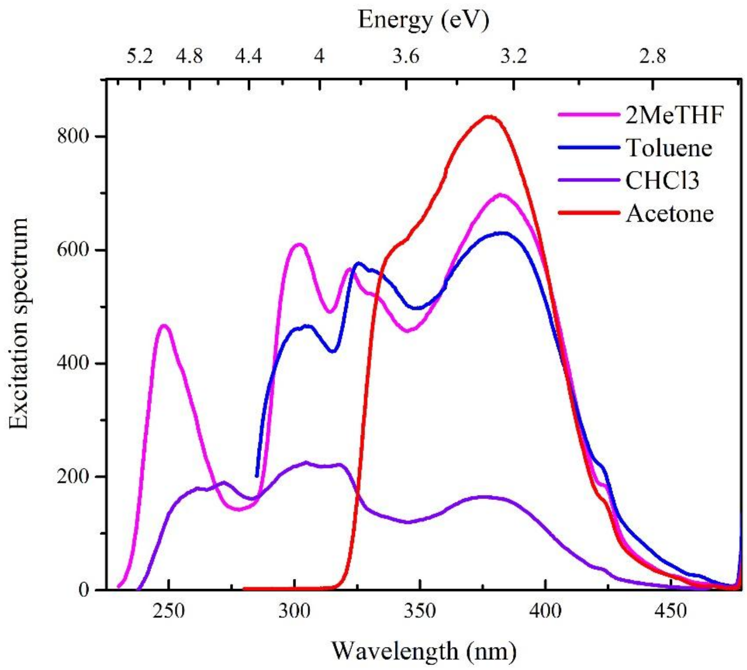

Figure S1.8: Excitation spectrum of **TD[7]Br** in different solvents at 298 K.

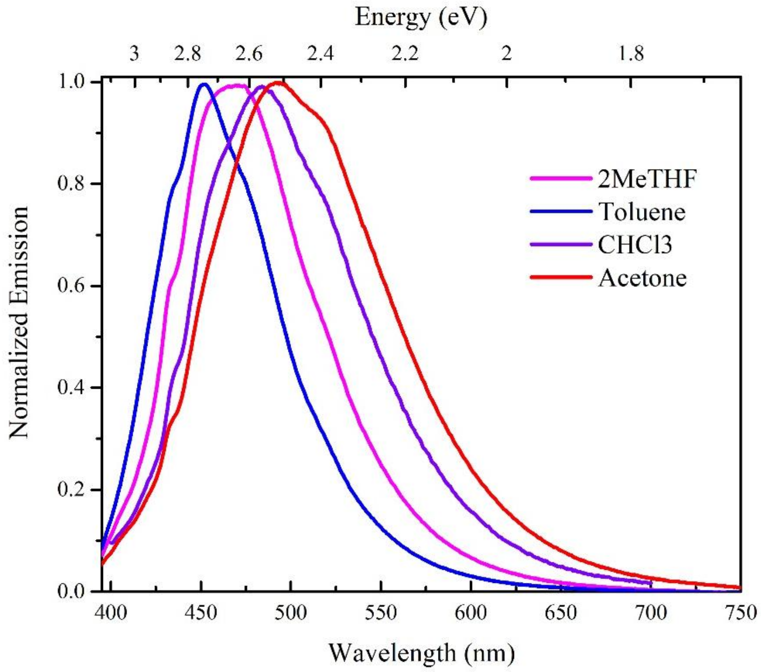

Figure S1.9: Emission spectrum of **TD[7]Br** in different solvents at 298 K.

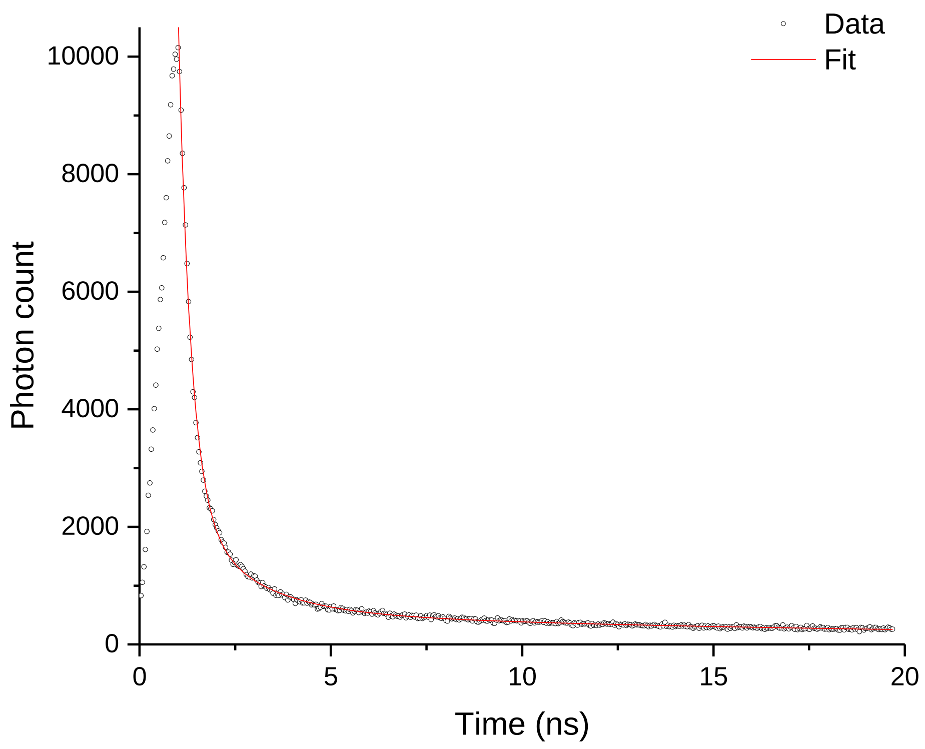

Figure S1.10: Results of the TCSPC experiment conducted on **[7]Br** in toluene at room temperature: the observed photon count (black dots), accurately modeled using a three-component exponential decay function (red line).

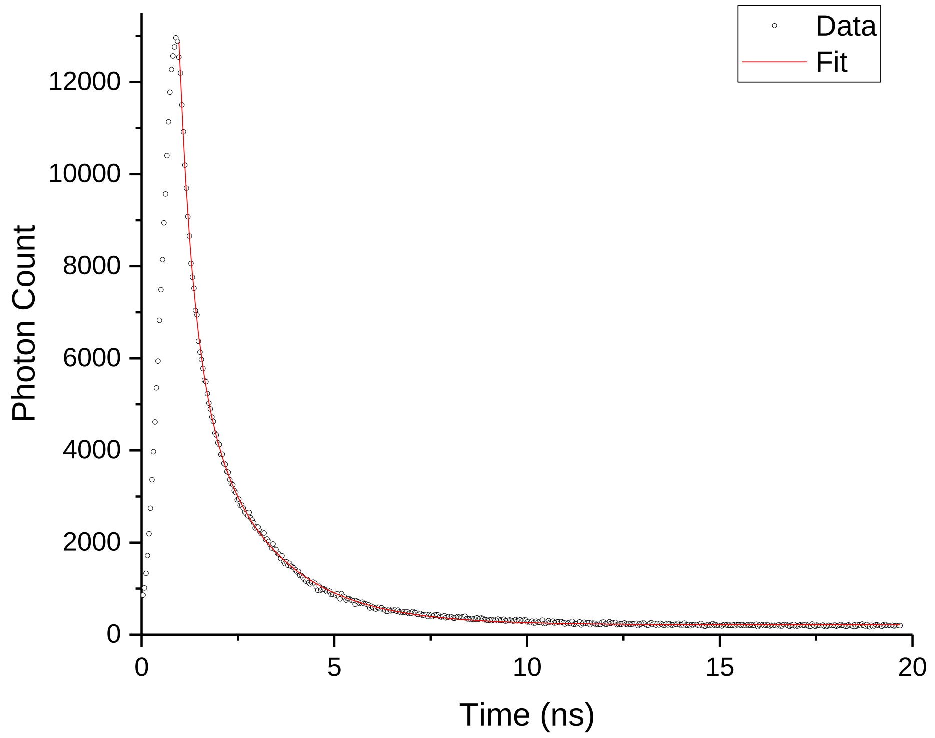

Figure S1.11: Results of the TCSPC experiment conducted on **TD[7]Br** in toluene at room temperature: the observed photon count (black dots), accurately modeled using a bi-component exponential decay function (red line).

1.7. References for experiments

1.1. Drexhage, K. H. Fluorescence Efficiency of Laser Dyes. *J. RES. NATL. BUR. STAN. SECT. A.* **1976**, *80A* (3), 421. https://doi.org/10.6028/jres.080A.044.

1.2. Sudhakar, A.; Katz, T. J. Directive Effect of Bromine on Stilbene Photocyclizations. an Improved Synthesis of [7]Helicene. *Tetrahedron Letters* **1986**, *27* (20), 2231–2234. https://doi.org/10.1016/S0040-4039(00)84494-9.

2. Computational part

2.1. Computational details

(*P*)-enantiomers of experimentally considered brominated helicenes were examined via calculations using density functional theory (DFT) and its time-dependent variant (TDDFT) methods. In all the computations no symmetry was explicitly imposed.

Ground-state and lowest-energy (S_1 and T_1) excited-state geometry optimizations along with optical rotation (OR) parameters, absorption and electronic circular dichroism (ECD) spectra calculations were performed with the Gaussian 16 (G16) program,[1] utilizing split-valence polarization SVP[2] basis set and employing the polarizable continuum model (PCM)[3,4,5,6,7] for toluene ($\varepsilon = 2.3741$) and tetrahydrofuran (THF, $\varepsilon = 7.4257$) with default G16 settings to account for solvent effects. THF was used to approximate 2-methyltetrahydrofuran solvent that was experimentally employed in low-temperature emission measurements but parameters of which are not implemented in G16. DFT ground-state geometry optimizations employed the B3LYP[8,9,10] exchange-correlation density functional. OR parameters, UV-vis and ECD spectra, and optimal S_1 and T_1 excited-state structures were calculated with TDDFT linear response approach using the PBE0[11,12] functional. For excited-state geometry optimizations, the Tamm-Dancoff[13,14,15,16,17,18] approximation to TDDFT (TDDFT-TDA) was used in addition to full TDDFT computations, as typically it enables to alleviate the problem of significant underestimation of T_1 energies modelled with full TDDFT, which has also be shown for some helicenic systems,[19,20,21,22,23] including the ones presented in these studies (see Table S2.5).

The OR parameters were computed at the sodium *D*-line wavelength $\lambda = 589.3$ nm. The UV-vis and ECD calculations reported here cover 120 lowest singlet excited states. The simulated UV-vis and ECD spectra shown were obtained as the sums of Gaussian functions (with the root mean square width of $\sigma = 0.2$ eV) centered at the vertical excitation energies and scaled using the calculated dipole or rotatory strengths.[24]

Luminescence dissymmetry factors (g_{lum}) for both singlet and triplet excited states were obtained based on TDDFT (for singlets) and TDDFT-TDA (for triplets) calculations of excitation energies performed at the respective G16-optimized S_1 and T_1 structures using the Amsterdam Density Functional (ADF) package from Amsterdam Modeling Suite, versions 2022.102 and 2023.101,[25,26] in the latter taking advantage of a recently developed code enabling computations of magnetic transition dipole moments and rotatory strengths for the triplet-singlet transitions.[22] In this part of the studies, the PBE0 functional, Slater-type all-electron double-zeta polarized DZP basis set,[27] conductor-like screening model of solvation (COSMO)[28] (toluene for S_1, THF for T_1), and the zeroth-order regular approximation (ZORA)[29,30,31,32] two-component relativistic Hamiltonian were employed (ZORA-SOC: ZORA with spin-orbit coupling). Center-of-nuclear-charge coordinates for excited-state structures were used to minimize origin-dependence problem of the length-gauge results.[33] Up to 6 lowest-energy singlet states and 12 lowest-energy triplet states (depending on the system) were cover in these computations to include as many as possible singlet states of sizable rotatory strength value; the energetic order of the excited states were thus first determined based on the

corresponding excitation energies calculations with ZORA applied as scalar correction (ZORA-SC: scalar ZORA).

Finally, to qualitatively assess SO-induced coupling between the electronic states and consequently suggest a possible relaxation pathway for the excited-state dynamics in **TD[7]Br**, TDDFT-TDA-PBE0//DZP COSMO(toluene) ZORA-SC calculations with perturbative inclusion of SOC[34] (ZORA-pSOC) were performed at the G16-optimized S_1 and T_1 structures (respectively TDDFT-PBE0//SVP PCM(toluene) and TDDFT-TDA-PBE0//SVP PCM(toluene)) using the ADF, version 2022.102, which enabled to obtain SOC strengths based on the matrix elements for the SO interaction between singlet and triplet states.

2.2. Additional computed results

Table S2.1: Selected dominant excitations and occupied (occ) – unoccupied (unocc) MO-pair contributions (greater than 10%) for (*P*)-**[7]Br**. Based on TDDFT-PBE0//SVP with continuum solvent model for toluene calculations. See Figure 6 in the main text for the corresponding simulated UV-vis and ECD spectra. See Figure S2.1 for MOs isosurfaces.

Excitation	E / eV	λ / nm	f	R / 10^{-40} esu^2 cm^2	occ no.	unocc no.	%
1	3.106	399	0.001	-0.94	116	117	54.1
					115	118	36.4
2	3.299	376	0.008	22.42	115	117	56.9
					116	118	35.6
3	3.343	371	0.197	801.65	116	118	52.7
					115	117	38.5
4	3.565	348	0.070	22.76	114	117	59.5
5	3.614	343	0.031	-39.07	115	118	41.2
					116	117	23.5
					114	117	15.3
					114	118	14.0
6	3.697	335	0.158	-194.52	114	118	71.3
					116	117	10.1
8	4.029	308	0.131	-54.01	113	118	30.3
					116	120	25.5
					115	119	21.5
					114	117	15.0
9	4.094	303	0.027	7.13	115	119	50.8
					113	118	44.2
10	4.127	300	0.272	-230.46	116	119	31.4
					113	117	29.9
					115	120	23.4

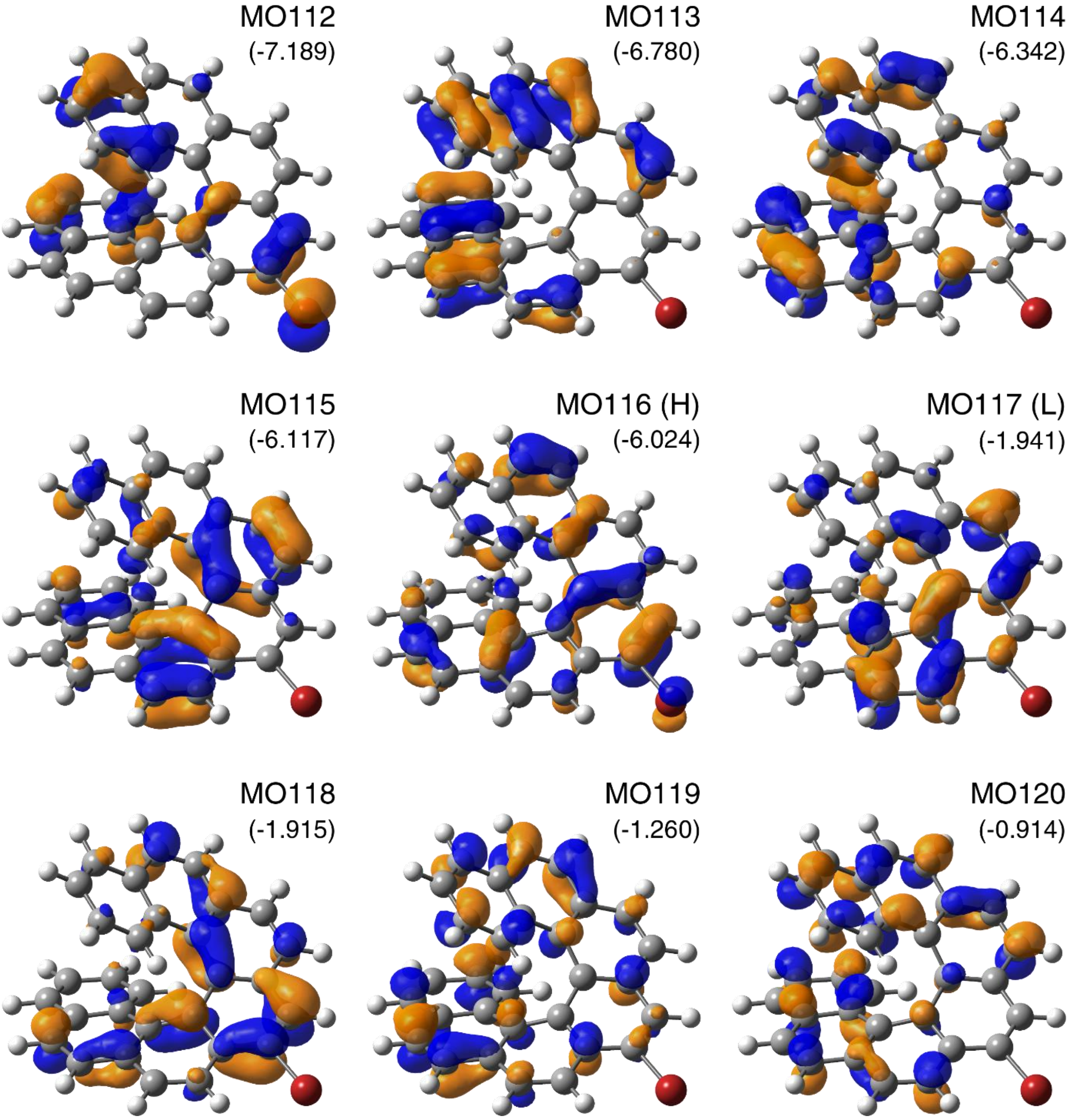

Figure S2.1: Isosurfaces (±0.04 au) of MOs involved in selected electronic transitions of **[7]Br**. Based on PBE0//SVP with continuum solvent model for toluene calculations. H = HOMO, L = LUMO. In parentheses, orbital energies in eV are listed.

Table S2.2: Selected dominant excitations and occupied (occ) – unoccupied (unocc) MO-pair contributions (greater than 10%) for (*P*)-**TD[7]Br**. Based on TDDFT-PBE0//SVP with continuum solvent model for toluene calculations. See Figure 6 in the main text for the corresponding simulated UV-vis and ECD spectra. See Figure S2.2 for MOs isosurfaces.

Excitation	E / eV	λ / nm	f	R / 10^{-40} esu^2 cm^2	occ no.	unocc no.	%
1	2.960	419	0.010	-11.41	118	119	80.2
					117	120	15.1
2	3.121	397	0.032	18.28	117	119	59.3
					118	120	37.0
3	3.152	393	0.174	602.15	118	120	59.5
					117	119	36.8
4	3.334	372	0.059	-88.79	117	120	71.9
					118	119	17.0
5	3.594	345	0.026	23.90	116	119	92.1
6	3.804	326	0.032	-4.19	116	120	85.7
					115	119	10.4
7	3.890	319	0.012	-13.82	118	121	45.1
					115	119	40.0
8	4.018	309	0.043	101.96	115	120	78.1
					117	121	13.8
9	4.080	304	0.741	-879.13	115	119	43.6
					118	121	31.1
10	4.129	300	0.201	-41.91	117	121	64.4
11	4.307	288	0.044	66.22	114	119	55.5
					112	119	12.1
12	4.344	285	0.155	170.22	118	122	62.5
					113	119	17.6

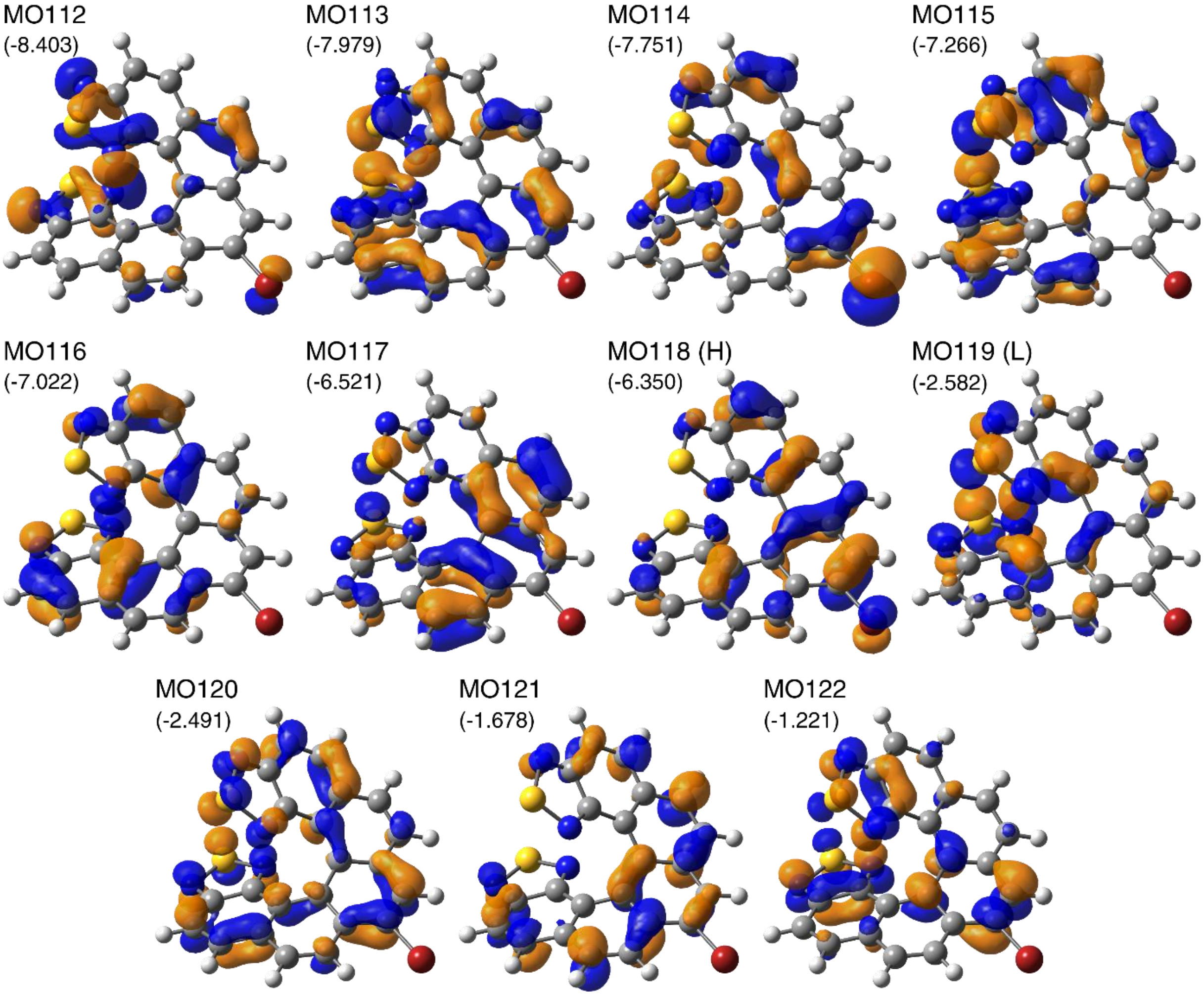

Figure S2.2: Isosurfaces (±0.04 au) of MOs involved in selected electronic transitions of **TD[7]Br**. Based on PBE0//SVP with continuum solvent model for toluene calculations. H = HOMO, L = LUMO. In parentheses, orbital energies in eV are listed.

Table S2.3: Selected dominant excitations and occupied (occ) – unoccupied (unocc) MO-pair contributions (greater than 10%) for (*P*)-**[6]Br**. Based on TDDFT-PBE0//SVP with continuum solvent model for toluene calculations. See Figure 6 in the main text for the corresponding simulated UV-vis and ECD spectra. See Figure S2.3 for MOs isosurfaces.

Excitation	E / eV	λ / nm	f	R / 10^{-40} esu^2 cm^2	occ no.	unocc no.	%
1	3.257	381	0.029	52.35	120	121	71.4
					119	122	24.9
3	3.629	342	0.374	897.82	119	122	71.5
					120	121	24.8
4	3.772	329	0.059	-102.73	120	122	43.3
					118	121	23.5
					119	121	22.3
5	3.954	314	0.221	-271.41	118	121	38.6
					120	122	25.6
					119	124	16.5
6	4.057	306	0.010	66.66	117	121	89.5
7	4.156	298	0.016	-19.60	120	123	60.4
					118	121	24.8
					117	122	12.4
8	4.185	296	0.025	6.52	118	122	56.1
					119	123	36.5
9	4.247	292	0.013	-5.82	119	123	40.0
					120	124	34.8
					118	122	21.8
10	4.414	281	0.062	-21.74	119	124	47.7
					117	122	45.5

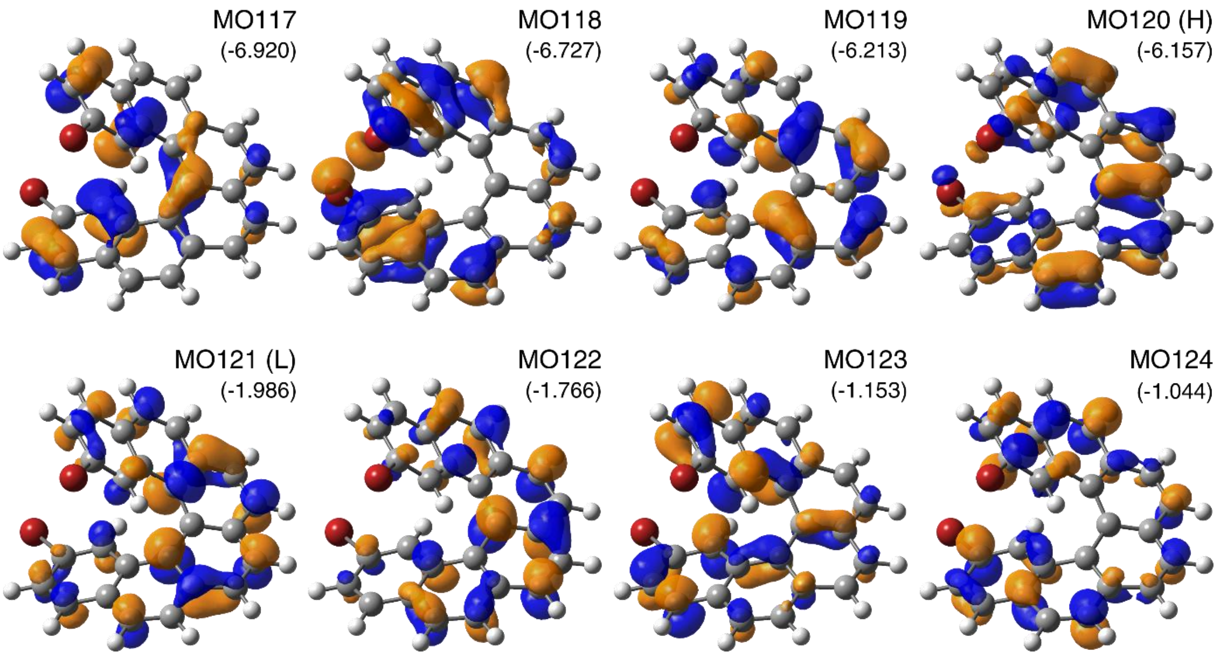

Figure S2.3: Isosurfaces (±0.04 au) of MOs involved in selected electronic transitions of **[6]Br**. Based on PBE0//SVP with continuum solvent model for toluene calculations. H = HOMO, L = LUMO. In parentheses, orbital energies in eV are listed.

Table S2.4: Experimental and calculated (TDDFT-PBE0//SVP with continuum solvent model for toluene) optical rotations (specific $[\alpha]_D$ in 10^{-1} deg cm^2 g^{-1} and molar rotation $[\phi]_D$ in 10^{-1} deg cm^2 mol^{-1}) for (*P*)-enantiomers of the considered brominated helicenes.

		**[7]Br**	**TD[7]Br**	**[6]Br**
Expt.	$[\alpha]_D$	2377	5984	–
	$[\phi]_D$	10872	28326	–
Calc.	$[\alpha]_D$	4677	4581	3958
	$[\phi]_D$	21393	21683	19245

Table S2.5: Computed emission data for the considered brominated helicenes as obtained via TDDFT(-TDA)-PBE0//SVP with continuum solvent (toluene and THF) model calculations: *E* (λ) – S_1-S_0 energy difference (in eV (in nm)) at TDDFT(-TDA) optimized S_1 geometry (for S_1) and T_1-S_0 energy difference (in eV (in nm)) at TDDFT(-TDA) optimized T_1 geometry (for T_1), corresponding to respectively fluorescence and phosphorescence energy; *f* – oscillator strength; *R* – rotatory strength, in cgs units of 10^{-40} esu^2 cm^2; L/H % – percentage contribution of LUMO→HOMO transition to the S_1→S_0 and T_1→S_0 emission (for S_1 and T_1, respectively).

	E (λ)	*f*	*R*	L/H %	*E* (λ)	*f*	*R*	L-H %
	toluene				THF			
(*P*)-**[7]Br**								
S_1^{TDDFT}	2.500 (496)	0.045	417.83	99.4	2.500 (496)	0.067	484.77	99.5
$S_1^{TDDFT\text{-}TDA}$	2.568 (483)	0.051	385.84	98.3	2.569 (483)	0.072	447.63	98.5
T_1^{TDDFT}	1.621 (765)	–	–	76.8†	1.625 (763)	–	–	76.9†
$T_1^{TDDFT\text{-}TDA}$	1.960 (632)	–	–	88.1	1.961 (632)	–	–	88.1
(*P*)-**TD[7]Br**								
S_1^{TDDFT}	2.294 (541)	0.010	182.20	97.5	2.387 (519)	0.024	295.20	97.9
$S_1^{TDDFT\text{-}TDA}$	not obtained				2.394 (518)	0.018	183.73	96.0
T_1^{TDDFT}	1.544 (803)	–	–	77.8	1.549 (800)	–	–	76.3
$T_1^{TDDFT\text{-}TDA}$	1.916 (647)	–	–	85.8	1.922 (645)	–	–	85.0
(*P*)-**[6]Br**								
S_1^{TDDFT}	3.051 (406)	0.033	61.13	73.9§	3.044 (407)	0.063	95.06	79.4§
$S_1^{TDDFT\text{-}TDA}$	3.074 (403)	0.023	29.18	67.0⸸	3.071 (404)	0.044	48.38	71.9⸸
T_1^{TDDFT}	1.846 (672)	–	–	66.9£	1.851 (670)	–	–	67.0£
$T_1^{TDDFT\text{-}TDA}$	2.236 (554)	–	–	83.6	2.240 (554)	–	–	83.7

† Additional MO-pair contribution: LUMO+1→HOMO-1: 13.4%

§ Additional MO-pair contribution: LUMO+1→HOMO-1: 22.9% / 17.5% for toluene / THF

⸸ Additional MO-pair contribution: LUMO+1→HOMO-1: 29.6% / 24.8% for toluene / THF

£ Additional MO-pair contribution: LUMO+1→HOMO-1: 19.4%

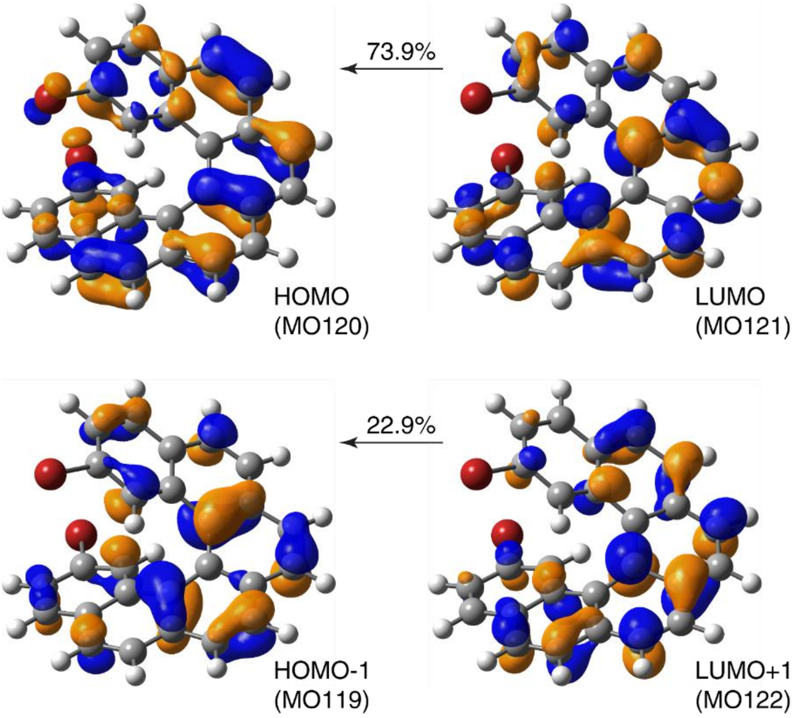

Figure S2.4: Dominant MO-pair contributions (isosurfaces: ±0.04 au) to $S_1 \rightarrow S_0$ transition for (*P*)-**[6]Br**. Based on TDDFT-PBE0//SVP PCM(toluene) calculations.

Table S2.6: Calculated (TDDFT(-TDA)-PBE0//DZP with continuum solvent model ZORA-SOC) emission data for the considered brominated helicenes ((*P*)-enantiomers): *E* – S_1-S_0 energy difference at TDDFT-PBE0//SVP/PCM(toluene)-optimized S_1 geometry (for S_1) and T_1-S_0 energy difference at TDDFT-TDA-PBE0//SVP/PCM(THF)-optimized T_1 geometry (for T_1), in eV, corresponding to respectively fluorescence and phosphorescence energy; $D = 3f/2E$ – dipole strength, in cgs units of 10^{-40} esu^2 cm^2; *R* – rotatory strength, in cgs units of 10^{-40} esu^2 cm^2; |**d**| – magnitude of electric transition dipole moment vector **d**, in SI units; |**m**| – magnitude of magnetic transition dipole moment vector **m**, in SI units; ∠(**d**,**m**) – angle between **d** and **m** vectors, in degree; $g_{lum} = 4R/D$ – emission dissymmetry factor; T_{11}, T_{12}, T_{13} – three triplet components of T_1 state.

System		*E*	*D*	*R*	|**d**|	|**m**|	∠(**d**,**m**)	g_{lum}
**[7]Br**	S_1	2.479	$5.82\cdot10^{4}$	$4.64\cdot10^{2}$	0.949	2.705	67	$3.19\cdot10^{-2}$
	T_{11}	1.946	$1.02\cdot10^{-3}$	$-4.64\cdot10^{-5}$	0.000	0.002	112	$-1.81\cdot10^{-1}$
	T_{12}	1.946	$2.92\cdot10^{-1}$	$-1.94\cdot10^{-3}$	0.002	0.004	117	$-2.65\cdot10^{-2}$
	T_{13}	1.946	$8.32\cdot10^{-2}$	$3.73\cdot10^{-4}$	0.001	0.004	80	$1.79\cdot10^{-2}$
**TD[7]Br**	S_1	2.342	$1.91\cdot10^{4}$	$2.52\cdot10^{2}$	0.543	2.079	62	$5.28\cdot10^{-2}$
	T_{11}	1.944	$4.85\cdot10^{-4}$	$-1.05\cdot10^{-5}$	0.000	0.001	106	$-8.65\cdot10^{-2}$
	T_{12}	1.944	$1.15\cdot10^{-1}$	$-1.14\cdot10^{-3}$	0.001	0.004	116	$-3.96\cdot10^{-2}$
	T_{13}	1.944	$4.99\cdot10^{-2}$	$-4.26\cdot10^{-4}$	0.001	0.004	106	$-3.41\cdot10^{-2}$
**[6]Br**	S_1	3.019	$4.22\cdot10^{4}$	$9.27\cdot10^{1}$	0.809	1.413	80	$8.78\cdot10^{-3}$
	T_{11}	2.240	$4.31\cdot10^{-3}$	$-6.97\cdot10^{-5}$	0.000	0.001	113	$-6.47\cdot10^{-2}$
	T_{12}	2.240	$1.04\cdot10^{-1}$	$2.22\cdot10^{-4}$	0.001	0.002	78	$8.52\cdot10^{-3}$
	T_{13}	2.240	$6.61\cdot10^{-3}$	$-1.30\cdot10^{-4}$	0.000	0.002	120	$-7.86\cdot10^{-2}$

Table S2.7: Selected SOC strengths (in cm^{-1}) computed based on the matrix elements for the SO interaction between singlet and triplet states of **TD[7]Br** obtained with TDDFT-TDA-PBE0//DZP with continuum solvent (toluene) model ZORA-pSOC calculations at the G16-optimized S_1 and T_1 structures (respectively TDDFT-PBE0//SVP PCM(toluene) and TDDFT-TDA-PBE0//SVP PCM(toluene)). In parentheses, the corresponding relative energy values (with respect to S_0, in eV) are provided.

@ S_1				@ T_1			
	T_1 (1.869)	T_2 (2.258)	T_3 (2.402)		T_1 (1.930)	T_2 (2.320)	T_3 (2.623)
S_0 (0.000)	13.53	14.21	7.00	S_0 (0.000)	4.92	8.11	8.29
S_1 (2.386)	6.11	4.18	7.09	S_1 (2.738)	3.97	5.14	2.99
T_1 (1.869)	–	10.66	10.11	T_1 (1.930)	–	9.08	3.90
T_2 (2.258)	–	–	10.86	T_2 (2.320)	–	–	2.38

Table S2.8: Computed emission data for (*P*)-**TD[7]Br** as obtained via TDDFT-TDA-PBE0//DZP with continuum solvent (toluene) model ZORA-SOC calculations: E (λ) – X_n-S_0 (X = S, T) energy difference (in eV (in nm)) at TDDFT-PBE0//SVP/PCM(toluene)-optimized S_1 geometry (at S_1) and at TDDFT-TDA-PBE0//SVP/PCM(toluene)-optimized T_1 geometry (at T_1); $D = 3f/2E$ – dipole strength, in cgs units of 10^{-40} esu^2 cm^2, for T_{1-3}: averaged over three triplet components; R – rotatory strength, in cgs units of 10^{-40} esu^2 cm^2, for T_{1-3}: averaged over three triplet components; $g_{lum} = 4R/D$ – emission dissymmetry factor, for T_{1-3}: based on averaged R and D; MO/MO: % – MO-pair contribution to emission transition, H = HOMO and L = LUMO, for MOs isosurfaces, see Figure S2.5.

	E (λ)	D	R	g_{lum}	MO/MO: %
S_1@S_1	2.386 (520)	$2.19\cdot10^{4}$	$2.11\cdot10^{2}$	$+3.86\cdot10^{-2}$	L/H: 96.1
T_2@S_1	2.258 (549)	$2.97\cdot10^{-1}$	$1.89\cdot10^{-3}$	$+2.54\cdot10^{-2}$	L/H-1: 61.6<br>L+1/H: 17.0
T_3@S_1	2.402 (516)	$5.71\cdot10^{1}$	$5.10\cdot10^{-1}$	$+3.57\cdot10^{-2}$	L+1/H: 55.5<br>L/H: 17.4
T_1@T_1	1.929 (643)	$4.26\cdot10^{-2}$	$-4.35\cdot10^{-4}$	$-4.09\cdot10^{-2}$	L/H: 85.8
T_2@T_1	2.319 (535)	$3.54\cdot10^{-1}$	$9.14\cdot10^{-4}$	$+1.03\cdot10^{-2}$	L+1/H: 75.5<br>L/H-2: 12.1
T_3@T_1	2.623 (473)	$1.66\cdot10^{0}$	$3.25\cdot10^{-3}$	$+7.85\cdot10^{-3}$	L/H-1: 82.1

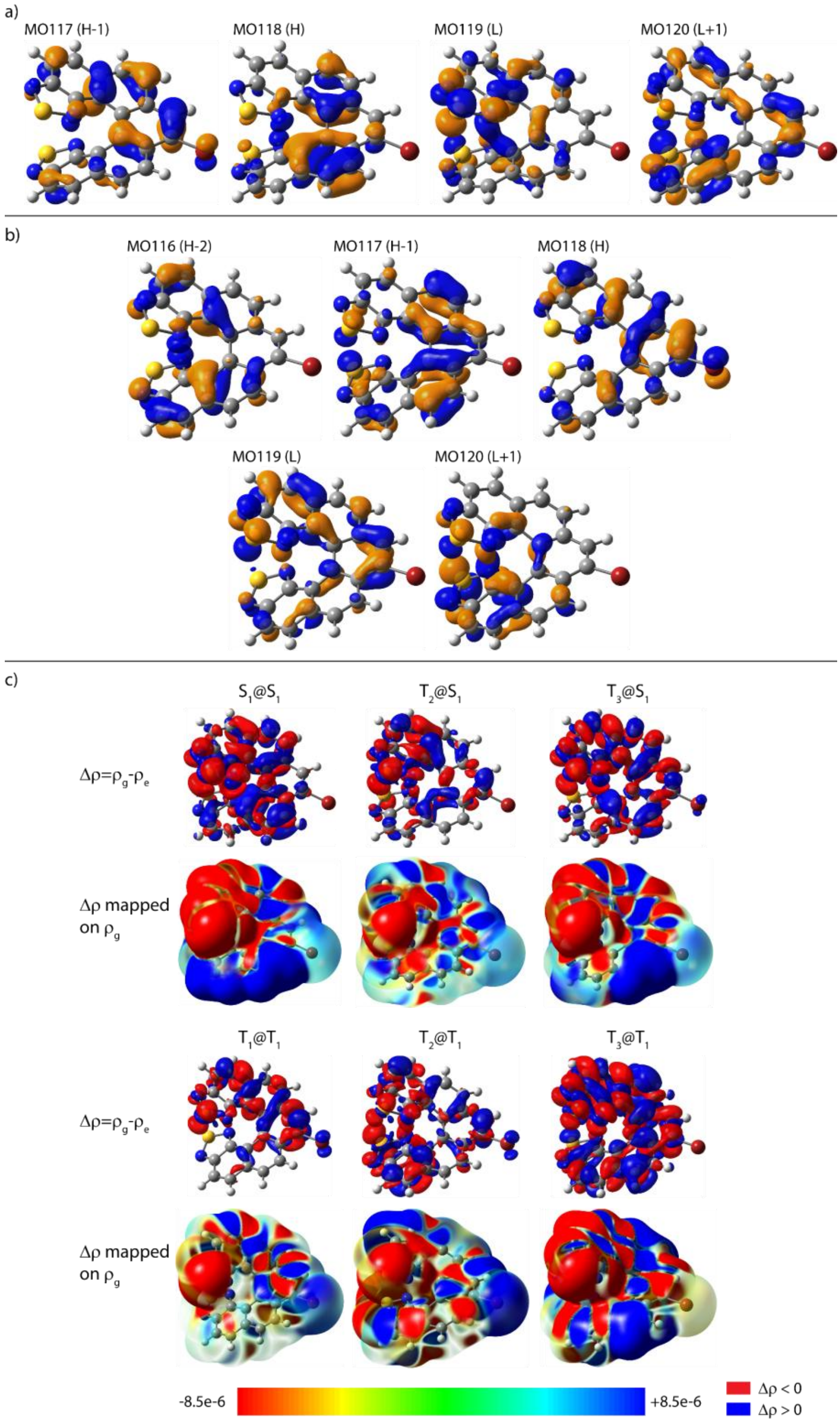

Figure S2.5: Isosurfaces (±0.04 au) of MOs involved in $X_n \rightarrow S_0$ emission transitions (X = S, T; n = 1, 2, 3) at S_1 (panel a) and at T_1 (panel b) structures of **TD[7]Br** listed in Table S2.8. H = HOMO, L = LUMO. Panel c: Isosurfaces (±0.0008 au) of the corresponding electron density differences between the S_0 ground state and the respective excited state, $\Delta\rho = \rho_g - \rho_e$, and isosurfaces (0.0004 au) of ρ_g color-mapped using the values of $\Delta\rho$. Electron density moves from the red region to the blue region when moving from the excited state to the ground state. Based on TDDFT-TDA-PBE0//SVP with continuum solvent (toluene) model calculations.

2.3. Cartesian coordinates for optimized structures

Optimized (DFT-B3LYP//SVP with continuum solvent (toluene) model) geometries of the studied systems along with the corresponding absolute energies:

The atomic symbol followed by three Cartesian coordinates, in Å.

(*P*)-**TD[7]Br**

Total energy = -4432.026507 au

C	-3.084267	1.709463	1.156571
C	-3.403797	2.945112	0.507481
H	-4.384991	3.397496	0.656846
C	-2.429424	3.535997	-0.237262
H	-2.616100	4.505298	-0.706828
C	-1.120750	2.950570	-0.432501
C	-0.125013	3.724985	-1.092618
H	-0.392235	4.710781	-1.480437
C	1.168636	3.279978	-1.149638
H	1.961724	3.916758	-1.548789
C	1.508009	1.978214	-0.682021
C	2.884532	1.604978	-0.605290
H	3.643258	2.326058	-0.913003
C	3.239690	0.403439	-0.065040
C	2.256006	-0.607676	0.217286
C	2.623748	-1.895394	0.699365
H	3.662783	-2.073818	0.975859
C	1.695951	-2.896725	0.812793
H	1.982021	-3.870021	1.218215
C	0.379250	-2.717798	0.308874
C	-0.512422	-3.857019	0.277004
H	-0.159539	-4.779201	0.745856
C	-1.735940	-3.823516	-0.318292
H	-2.399907	-4.688489	-0.346024
C	-2.119472	-2.611551	-0.978006
C	-1.267495	-1.429700	-0.924127
C	-0.011336	-1.445501	-0.187171
C	0.877116	-0.316039	-0.051249
C	0.491626	1.081274	-0.237295
C	-0.799666	1.654380	0.056706
C	-1.793544	1.065950	0.943317
N	-3.867394	1.070496	2.022508
N	-1.644054	-0.025836	1.688061

S	-3.024527	-0.238387	2.554608
N	-1.742026	-0.430633	-1.662299
N	-3.212382	-2.453424	-1.720651
S	-3.157234	-0.927874	-2.334510
Br	5.101243	0.059730	0.213431

(*P*)-**[7]Br**

Total energy = -3726.525296 au

Br	5.079906	-0.024472	0.170158
C	-2.388342	-0.616870	2.836149
H	-2.086713	-1.416441	3.517285
C	-3.724325	-0.162949	2.830383
H	-4.466317	-0.627806	3.484332
C	-4.072703	0.901660	2.022352
H	-5.089693	1.302751	2.042926
C	-3.117122	1.526281	1.178918
C	-1.784280	1.004230	1.100522
C	-1.448935	-0.048489	1.993794
H	-0.420676	-0.398237	2.047968
C	-3.455007	2.721971	0.467789
H	-4.480774	3.095638	0.523122
C	-2.490361	3.425739	-0.192041
H	-2.722260	4.387738	-0.656583
C	-1.150763	2.927946	-0.299667
C	-0.819052	1.640293	0.210187
C	-0.143697	3.749306	-0.893086
H	-0.424677	4.738476	-1.263291
C	1.160756	3.341049	-0.910694
H	1.954878	4.007574	-1.255939
C	1.504709	2.014177	-0.509542
C	0.485502	1.092168	-0.126564
C	2.874985	1.621063	-0.478960
H	3.640956	2.355924	-0.731450
C	3.219090	0.366395	-0.058697
C	2.227714	-0.652201	0.140572
C	0.852997	-0.319509	-0.099278
C	2.569453	-1.975212	0.553283
H	3.608071	-2.195546	0.799853
C	1.605959	-2.938146	0.672528
H	1.858473	-3.929151	1.057829
C	0.279925	-2.700652	0.204639
C	-0.072286	-1.422536	-0.310665

C	-0.681155	-3.763577	0.229259
H	-0.393886	-4.708640	0.697495
C	-1.922599	-3.603989	-0.312774
H	-2.660284	-4.409382	-0.268046
C	-2.248053	-2.410105	-1.033225
C	-1.306571	-1.330106	-1.084362
C	-3.462655	-2.324954	-1.762527
H	-4.175350	-3.150532	-1.684643
C	-3.728099	-1.247023	-2.584324
H	-4.661764	-1.198688	-3.150188
C	-2.761172	-0.228651	-2.720387
H	-2.932660	0.597088	-3.415371
C	-1.586602	-0.271609	-1.989755
H	-0.847878	0.511542	-2.143813

(*P*)-**[6]Br**

Total energy = -6146.270230 au

C	-1.524808	2.850614	1.495540
C	-0.240081	2.708257	0.911119
C	0.384718	1.419646	0.877602
C	-0.273441	0.354306	1.547676
C	-1.507891	0.542985	2.139662
C	-2.168531	1.787800	2.097231
C	0.464738	3.853835	0.421692
C	1.768649	3.742571	0.036583
C	2.427935	2.469652	-0.000250
C	1.698487	1.273285	0.256691
C	3.834318	2.415450	-0.238819
C	4.503501	1.227410	-0.136728
C	3.788010	0.000000	0.000003
C	2.356984	-0.000000	0.000003
C	4.503501	-1.227409	0.136734
C	3.834319	-2.415450	0.238821
C	2.427937	-2.469652	0.000252
C	1.698488	-1.273285	-0.256686
C	1.768650	-3.742571	-0.036582
C	0.464740	-3.853836	-0.421690
C	-0.240080	-2.708257	-0.911118
C	0.384718	-1.419646	-0.877601
C	-1.524805	-2.850615	-1.495541
C	-2.168528	-1.787800	-2.097235
C	-1.507888	-0.542987	-2.139664

C	-0.273438	-0.354307	-1.547676
Br	-2.342063	-0.911327	3.058082
Br	-2.342058	0.911327	-3.058085
H	0.207884	0.617024	-1.627078
H	-1.997745	-3.836076	-1.487203
H	-3.151613	-1.908929	-2.554226
H	2.342596	-4.628519	0.246974
H	-0.036644	-4.824833	-0.429063
H	5.594361	-1.191752	0.194026
H	4.372677	-3.348803	0.420914
H	4.372675	3.348804	-0.420912
H	5.594360	1.191754	-0.194020
H	-0.036646	4.824832	0.429064
H	2.342594	4.628519	-0.246973
H	0.207882	-0.617025	1.627076
H	-3.151618	1.908925	2.554220
H	-1.997748	3.836076	1.487198

2.4. References for computations

[1] Gaussian 16, Revision C.01, M. J. Frisch, G. W. Trucks, H. B. Schlegel, G. E. Scuseria, M. A. Robb, J. R. Cheeseman, G. Scalmani, V. Barone, G. A. Petersson, H. Nakatsuji, X. Li, M. Caricato, A. V. Marenich, J. Bloino, B. G. Janesko, R. Gomperts, B. Mennucci, H. P. Hratchian, J. V. Ortiz, A. F. Izmaylov, J. L. Sonnenberg, D. Williams-Young, F. Ding, F. Lipparini, F. Egidi, J. Goings, B. Peng, A. Petrone, T. Henderson, D. Ranasinghe, V. G. Zakrzewski, J. Gao, N. Rega, G. Zheng, W. Liang, M. Hada, M. Ehara, K. Toyota, R. Fukuda, J. Hasegawa, M. Ishida, T. Nakajima, Y. Honda, O. Kitao, H. Nakai, T. Vreven, K. Throssell, J. A. Montgomery, Jr., J. E. Peralta, F. Ogliaro, M. J. Bearpark, J. J. Heyd, E. N. Brothers, K. N. Kudin, V. N. Staroverov, T. A. Keith, R. Kobayashi, J. Normand, K. Raghavachari, A. P. Rendell, J. C. Burant, S. S. Iyengar, J. Tomasi, M. Cossi, J. M. Millam, M. Klene, C. Adamo, R. Cammi, J. W. Ochterski, R. L. Martin, K. Morokuma, O. Farkas, J. B. Foresman, and D. J. Fox, Gaussian, Inc., Wallingford CT, 2016.

[2] F. Weigend, R. Ahlrichs, *Phys. Chem. Chem. Phys.* **2005**, *7*, 3297–3305.

[3] J. Tomasi, B. Mennucci, R. Cammi, *Chem. Rev.* **2005**, *105*, 2999–3094.

[4] M. Cossi, V. Barone, R. Cammi, J. Tomasi, *Chem. Phys. Lett.* **1996**, *255*, 327–335.

[5] V. Barone, M. Cossi, *J. Phys. Chem. A* **1998**, *102*, 1995–2001.

[6] M. Cossi, V. Barone, *J. Chem. Phys.* **2001**, *115*, 4708–4717.

[7] G. Scalmani, M. J. Frisch, *J. Chem. Phys.* **2010**, *132*, 114110.

[8] A. D. Becke, *J. Chem. Phys.* **1993**, *98*, 5648–5652.

[9] C. Lee, W. Yang, R. G. Parr, *Phys. Rev. B*, **1988**, *37*, 785–789.

[10] P. J. Stephens, F. J. Devlin, C. F. Chabalowski, M. J. Frisch, *J. Phys. Chem.* **1994**, *98*, 11623–11627.

[11] C. Adamo, V. Barone, *J. Chem. Phys.* **1999**, *110*, 6158–6169.

[12] M. Ernzerhof, G. E. Scuseria, *J. Chem. Phys.* **1999**, *110*, 5029–5036

[13] I. Tamm, *J. Phys. USSR* **1945**, *9*, 449–460.

[14] S. M. Dancoff, *Phys. Rev.* **1950**, *78*, 382–385.

[15] S. Hirata, M. Head-Gordon, *Chem. Phys. Lett.* **1999**, *314*, 291–299.

[16] M. J. G. Peach, M. J. Williamson, D. J. Tozer, *J. Chem. Theory Comput.* **2011**, *7*, 3578–3585.

[17] M. J. G. Peach, D. J. Tozer, *J. Phys. Chem. A* **2012**, *116*, 9783–9789.

[18] M. J. G. Peach, N. Warner, D. J. Tozer, *Mol. Phys.* **2013**, *111*, 1271–1274.

[19] C. Shen, E. Anger, M. Srebro, N. Vanthuyne, K. K. Deol, T. D. Jefferson, G. Muller, J. A. G. Williams, L. Toupet, C. Roussel, J. Autschbach, R. Reau, J. Crassous, *Chem. Sci.* **2014**, *5*, 1915–1927.

[20] E. Anger, M. Srebro, N. Vanthuyne, C. Roussel, L. Toupet, J. Autschbach, R. Reau, J. Crassous, *Chem. Commun.* **2014**, *50*, 2854–2856.

[21] N. Saleh, M. Srebro, T. Reynaldo, N. Vanthuyne, L. Toupet, V. Y. Chang, G. Muller, J. A. G. Williams, C. Roussel, J. Autschbach, J. Crassous, *Chem. Commun.* **2015**, *51*, 3754–3757.

[22] H. D. Ludowieg, M. Srebro-Hooper, J. Crassous, J. Autschbach, *ChemistryOpen* **2022**, *11*, e202200020.

[23] K. Dhbaibi, P. Morgante, N. Vanthuyne, J. Autschbach, L. Favereau, J. Crassous, *J. Phys. Chem. Lett.* **2023**, *14*, 1073-1081.

[24] J. Autschbach, T. Ziegler, S. J. A. van Gisbergen, E. J. Baerends, *J. Chem. Phys.* **2002**, *116*, 6930-6940.

[25] G. te Velde, F. M. Bickelhaupt, E. J. Baerends, C. Fonseca Guerra, S. J. A. van Gisbergen, J. G. Snijders, T. Ziegler, *J. Comput. Chem.* **2001**, *22*, 931-967.

[26] ADF 2022.1 and ADF 2023.1, SCM, Theoretical Chemistry, Vrije Universiteit, Amsterdam, The Netherlands, http://www.scm.com.

[27] E. van Lenthe, E. J. Baerends, *J. Comput. Chem.* **2003**, *24*, 1142-1156.

[28] C. C. Pye, T. Ziegler, *Theor. Chem. Acc.* **1999**, *101*, 396-408.

[29] E. van Lenthe, E. J. Baerends, J. G. Snijders, *J. Chem. Phys.* **1993**, *99*, 4597-4610.

[30] E. van Lenthe, E. J. Baerends, J. G. Snijders, *J. Chem. Phys.* **1994**, *101*, 9783-9792.

[31] A. Rosa, E. J. Baerends, S. J. A. van Gisbergen, E. van Lenthe, J. A. Groeneveld, J. G. Snijders, *J. Am. Chem. Soc.* **1999**, *121*, 10356-10365.

[32] F. Wang, T. Ziegler, E. van Lenthe, S. J. A. van Gisbergen, E. J. Baerends, *J. Chem. Phys.* **2005**, *122*, 204103.

[33] F. Gendron, B. Moore II, O. Cador, F. Pointillart, J. Autschbach, B. Le Guennic, *J. Chem. Theory Comput.* **2019**, *15*, 4140-4155.

[34] F. Wang, T. Ziegler, *J. Chem. Phys.* **2005**, *123*, 154102.
